# A new exposure protocol adapted for wild bees reveals species-specific impacts of the sulfoximine insecticide sulfoxaflor

**DOI:** 10.1007/s10646-024-02750-2

**Published:** 2024-04-22

**Authors:** Justine Dewaele, Alexandre Barraud, Sara Hellström, Robert J. Paxton, Denis Michez

**Affiliations:** 1 Research Institute for Biosciences, Laboratory of Zoology, University of Mons (UMons), Place du Parc 20, 7000 Mons, Belgium; 2 Univ. Lille, CNRS, UMR 8198 – Evo-Eco-Paleo, F-59000 Lille, France; 3 Pollinis, 10 rue Saint-Marc, 75002 Paris, France; 4 General Zoology, Institute for Biology, Martin Luther University Halle-Wittenberg, Hoher Weg 8, 06120 Halle, Germany

**Keywords:** Comparative ecotoxicology, *Bombus*, *Osmia*, Pollinators, Pesticides, Non-Apis bees

## Abstract

Wild bees are crucial pollinators of flowering plants and concerns are rising about their decline associated with pesticide use. Interspecific variation in wild bee response to pesticide exposure is expected to be related to variation in their morphology, physiology, and ecology, though there are still important knowledge gaps in its understanding. Pesticide risk assessments have largely focussed on the Western honey bee sensitivity considering it protective enough for wild bees. Recently, guidelines for *Bombus terrestris* and *Osmia bicornis* testing have been developed but are not yet implemented at a global scale in pesticide risk assessments. Here, we developed and tested a new simplified method of pesticide exposure on wild bee species collected from the field in Belgium. Enough specimens of nine species survived in a laboratory setting and were exposed to oral and topical acute doses of a sulfoximine insecticide. Our results confirm significant variability among wild bee species. We show that *Osmia cornuta* is more sensitive to sulfoxaflor than *B. terrestris*, whereas *Bombus hypnorum* is less sensitive. We propose hypotheses on the mechanisms explaining interspecific variations in sensitivity to pesticides. Future pesticide risk assessments of wild bees will require further refinement of protocols for their controlled housing and exposure.

## Introduction

Animals are pollen vectors for more than 85% of angiosperms (Ollerton et al. 2011). Many animal groups are described as pollinators: butterflies, flies, beetles, wasps, bats, birds, lizards, and mammals (Buchmann and Nabhan 1997). Bees are particularly important because they rely almost exclusively on floral resources (i.e. pollen and nectar) for food, both as adults and larvae (Michener 2007). With more than 20,000 species recorded worldwide (Michener 2007), and more than 2000 species recorded in Europe (Ghisbain et al. 2023) wild bee species exhibit significant variability in terms of phenology, host-plant use, nesting behavior, sociality and body size (Danforth et al. 2013; Michener 2007; Michez et al. 2019).

Losses and declines in managed and wild populations of bees have been reported worldwide (Cameron et al. 2011; Duchenne et al. 2020; Goulson et al. 2015). The major drivers have been well-identified in Europe (Nieto et al. 2014). Agricultural intensification notably associated with habitat loss (e.g. Persson et al. 2015; Vray et al. 2019) and pesticide use (Goulson et al. 2015) seems to play a key role (Dicks et al. 2021). As bees forage on flowering crops and adjacent flowers, they may be frequently exposed to pesticides (Godfray et al. 2014), either by contact exposure during pesticide application while foraging on flowers or collecting nesting material, or by oral exposure through the consumption of pesticide-treated floral resources (i.e. pollen and nectar) (e.g. Krupke et al. 2012). Yet the relative importance of these agrochemicals in the decline remains unclear (Goulson et al. 2015; Johnson and Corn 2015), with most studies based either on correlative analyses between the use of pesticides and the population decline (e.g. Woodcock et al. 2016) or on meta-analyses summarizing the results of diverse experimental protocols on different species (Arena and Sgolastra 2014). So far, less than 20 wild bee species, including two honey bee species (i.e. genus *Apis*) and four bumble bee species (i.e. genus *Bombus*), have been successfully kept under laboratory conditions (Arena and Sgolastra 2014; Helson et al. 1994; Leonard and Harmon-Threatt 2019; Scott-Dupree et al. 2009; Tadei et al. 2022; Thompson 2001). Full dose-response experiments are usually not possible because of the difficulty in obtaining enough individuals, the relatively short lifespan of solitary bees (around 14 days for species like *Andrena vaga* and *Anthophora plumipes* compared to 21 days for the western honey bee *Apis mellifera*; (Prado et al. 2020; Straka et al. 2014) and the high mortality of control groups.

Therefore, experimental methods currently used to assess the toxicity of pesticides to bees mainly rely, at least for initial tests, on determining acute toxicity through dose-response experiments (i.e. median lethal dose, LD50) on workers of *A. mellifera* (EFSA et al. 2020; OECD 2017a, 1998). This species is domesticated, easy to breed and to maintain under laboratory conditions. They are usually kept as a group of 10 or more individuals in a standardized cage (Williams et al. 2013) or in Nicot® cages when isolating individuals, held in a controlled rearing room (temperature at 33 °C, relative humidity between 50 and 70%) and under constant darkness (Franklin and Raine 2019; OECD 1998b, 1998a, 2017a). Yet, considering the ecological, physiological and morphological variability among wild bee species, the current use of *A. mellifera* sensitivity to predict hazards of pesticides for wild bees could lead to a considerably biased estimation of the adverse effects of pesticides on wild bees (Arena and Sgolastra 2014; Rundlöf et al. 2015; Uhl et al. 2016; Wood et al. 2020). Interspecific variations in the sensitivity of wild bee species have been highlighted through meta-analysis (Arena and Sgolastra 2014), in which data related to LC50s (concentration at which 50% of individuals die) for different species were compared. It appeared that 95% of bees were less than a factor of 10 different from honeybees in their sensitivity to pesticides, but there were outliers in both directions. Wide variation in size and body weight among wild bee species could be related to variation in their sensitivity to pesticides. Sensitivity has been shown to increase with the body surface-to-volume ratio (Johansen 1972; Pamminger 2021; Uhl et al. 2016).

The European Food Safety Authority (EFSA) suggested in 2013, and more recently in their revised guidance document on the risk assessment of plant protection products on bees, to include two other model species in pesticide risk assessment (EFSA 2013; EFSA et al. 2023): the Buff-tailed Bumble bee *Bombus terrestris* and Mason bee of the genus *Osmia*. Standard protocols for acute toxicity protocols have been developed for *B. terrestris* (OECD 2017b, 2017c) and are under development for Osmia spp. (EFSA et al. 2023; Medrzyck et al. 2021; Spurgeon et al. 2016). However, these species share some key characteristics with *A. mellifera* that could be involved in the variation in sensitivity among wild bees, such as their size (e.g. same or bigger) and lectism (e.g. pollen generalist species). Therefore, a great part of the actual wild bee diversity and sensitivity could be disregarded (Ghisbain 2021; Sgolastra et al. 2019).

Understanding variation in pesticide sensitivity among bee species in the context of bee decline and agricultural intensification is necessary yet challenging. The wide diversity of wild bee species and the lack of knowledge on their ecological and physiological traits as well as on their survivability under laboratory conditions makes it important to develop new protocols and identify their differences with current model species. In this study, we aim to (i) test a protocol adjusted from OECD conditions to maintain bees alive in laboratory conditions when collected from the wild; (ii) compare the effects on individual mortality of oral and topical acute exposure to sulfoxaflor, a neonicotinoid-like insecticide that was recently banned for outdoor use in EU due to risks towards invertebrates; and (iii) assess factors that could explain the interspecific variation in sensitivity to insecticides. We expected to find species-specific challenges for laboratory acclimation and interspecific variation in sensitivity to sulfoxaflor exposure (Arena and Sgolastra 2014), probably related to body size.

## Material and methods

### Bee species, selection and sampling

First, we selected 17 common non-endangered wild bee species in Belgium to perform the first housing assay (Drossart et al. 2019). The Buff-tailed Bumble bee *B. terrestris* was considered here as the reference domesticated species whose annual colonies can have a large number of workers (>500), and are commercially available (Rasmont et al. 2008). Commercial queen-right colonies each containing ca. 100 *B. terrestris* workers (Biobest BV, Waterloo, Belgium), were maintained in a dark room at 25 ± 5 °C and 60 ± 5% humidity (Tasei and Aupinel 2008). They were fed ad libitum with 50% w/w sugar sirup and once a week with 10 g of freeze-dried *Salix* spp. pollen per colony.

For 15 of the 17 other bee species, females were sampled from the field (Table 1). Males were not considered as they are much more difficult to capture in large numbers. We targeted the following species: two Andrenidae species, *A. fulva* and *A. vaga*; three Colletidae species, *Colletes daviesanus, Colletes hederae* and *Hylaeus signatus*; one Halictidae species, *Halictus scabiosae*; one Melittidae species, *Dasypoda hirtipes*; four Apidae species, *A. plumipes*, *B. lapidarius*, *B. hypnorum* and *B. pascuorum;* six Megachilidae species, *Anthidium manicatum*, *Chelostoma florisomne*, *Heriades truncorum*, *Osmia caerulescens*, *Osmia cornuta*, and *Osmia leaiana* (Table 1).

**Table 1:** Morphological and ecological characteristics of tested species

The gray sections show the species that were not included in the exposure experiments because of: (i) a low number of collected individuals, (ii) low survival in the laboratory or (iii) a low number of individuals feeding during the oral experiment. The number of individuals included in the oral experiment is the number of individuals able to feed from the cuvette. N = Number of individuals collected from the field. P = Pollen generalist (i.e., species foraging on pollen from more than one plant family). O = Pollen specialist (i.e., species foraging on pollen from one plant family only). PS = primitively social. S = solitary (Michez et al. 2019)

**Fig. 1:**
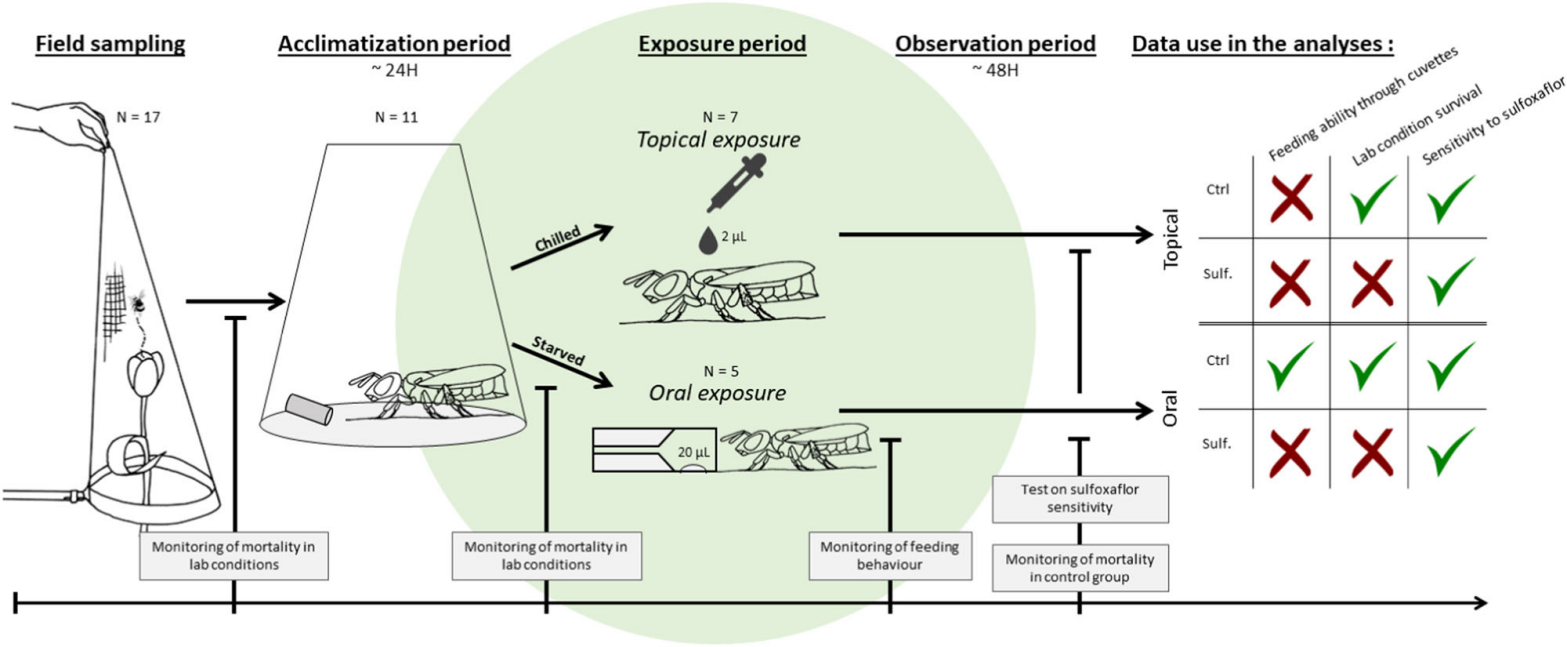
Schematic representation of the experimental procedure. N Number of species at each step. Ctrl Control group. Sulf. Group exposed to sulfoxaflor

^a^Species tested in Nicot cages with syringe-feeders. Other species were tested following our new protocol (Fig. 1)

All species except *A. plumipes* and *B. hypnorum* were collected as foraging adults using an insect hand-net in their natural habitats around the city of Mons (Belgium). These habitats were not located in agricultural areas, so the bees were less likely to be previously exposed to pesticides. However, all the selected bee species can be found foraging in and near orchards, crops or crop edges (Fiordaliso et al. 2022), and could all be exposed to pesticides during their lifecycle through for example, spray drift, direct exposure during crop spraying or contact exposure through soil for ground-nesting species.

To collect *A. plumipes*, clay blocks were set out in April in a pesticide-free garden area in Halle (Germany); the clay blocks were readily occupied by female *A. plumipes*. The clay blocks were then brought into the laboratory in September, from which brood cells were carefully excavated by hand. The brood cells containing live adults were then overwintered at 4 °C. At the beginning of experiments in the following April, brood cells were transferred to a 21 °C incubator and emergence was checked daily. Emerged adults were then transferred back to 4 °C until sufficient animals had emerged to populate an experiment (maximum four days).

*B. hypnorum* workers were obtained from wild colonies nesting in artificial structures, similar to bird’s nest (see Przybyla et al. (2023) for protocol details), from a private garden (Belgium, Luxembourg) and brought back to the laboratory.

### Housing, acclimation and feeding abilities

Before the beginning of the experiments, each bee was weighed to the nearest milligram, placed individually under an inverted see-through plastic beaker (Fig. 1), and fed *ad libitum* with 50% w/w sugar solution through soaked cotton capillaries (OECD 2017b, 2017c) for one day of acclimation. An alternative feeding method was tested: the use of 2 mL BD Emerald™ plastic syringes with 40 µL tips cut off to enlarge the feeding hole for bees as suggested by OECD guidelines (OECD 2017c). However, apart from the three bumble bee species, none of the wild bees successfully fed from them.

During the duration of the experiment, bumble bee species were kept under constant darkness, and all other species were under the prevailing natural light regime. Bumble bee species were kept at the same temperature and humidity conditions as the *B. terrestris* colonies (i.e., 25 ± 5 °C and 60 ± 5% humidity), while the other bees were kept at room temperature (i.e., *ca* 21 °C). For each species, individuals were sorted into to ensure a similar mean body size across treatment groups.

### Sulfoxaflor exposure

The experimental setups for topical and oral exposure were adapted from the OECD guidelines (OECD 2017b, 2017c; See Supplementary Tables S1 and S2 for adjustment details), and the improved protocols for testing agrochemicals on bees (Medrzyck et al. 2021), as a first attempt to expose eight species (Table 1; see Section 3.1) following the OECD guidelines showed that the wild bees cannot survive for long in nicot cages, or do not easily feed from the syringes. Specimens were exposed to sulfoxaflor individually (Fig. 1). The OECD guidelines recommend the use of at least 30 individuals for each treatment group. However, since the majority of wild bee species are not commercially available, and therefore not available in large numbers, for some species we used fewer individuals (Table 1).

#### Sulfoxaflor and control treatments

All the species in which sufficient (N > 10 individuals per species) specimens survived the acclimation phase (see below, Fig. 1) were exposed to lethal doses of sulfoxaflor either orally at 0.563 μg/g body weight or topically at 10.4 μg/bee, doses higher than the published median lethal doses for *B. terrestris* (for oral LD50 0.027 µg/bee and for contact LD50 7.55 µg/bee published by EFSA 2014; for oral LD50 0.126 µg/bee and for contact LD50 6.322 µg/bee published by Linguadoca et al. 2022). These values were calculated from unpublished preliminary data. While it was possible to prepare individual solutions of sulfoxaflor concentrations depending on the bee weight with precision for the oral exposure protocol, individuals exposed topically, received the same sulfoxaflor amount without taking their mass into account. Indeed, as bees were exposed through a droplet of 2 µL applied on the thorax, preparing treatment solutions and exposing each individual to the same sulfoxaflor concentration seemed more precise than preparing individual treatment solutions with different concentrations for each individual (see Supplementary Tables S3 and S4 for dose details).

A positive control experiment using dimethoate was performed on the model species *B. terrestris* following the same oral and topical exposure protocols we used for wild bees, with a dose of 10 μg/bee dimethoate (OECD 2017b, 2017c). All the bees exposed to dimethoate died, confirming the suitability of the adapted protocol to detect sensitivity in bees (See Supplementary Table S5 for detailed results).

To prepare all sulfoxaflor (CAS n°946578-00-3, Greyhound Chromatography and Allied Chemicals) and dimethoate (PESTANAL^®^, Sigma-Aldrich) treatments, stock solutions were first prepared by diluting in acetone. Then, a final dilution in distilled water (for topical exposure experiments) or 50% w/w sugar-water solution (for oral exposure experiments) was performed to achieve the aimed concentrations without exceeding a 5% acetone concentration (OECD 2017c). The prepared treatment and control solutions were used directly after preparation.

#### Oral exposure

After a 12-hour acclimation period (Fig. 1), the sugar-soaked capillaries were removed from the see-through plastic beakers and the bees starved for four hours. This was performed to achieve a uniform hunger level across all individuals and ensure a standard feeding process. Then, for each treatment group, a 20 μL droplet of either treatment (0.563 μg/g b.w sulfoxaflor) or control solution (i.e., 50% w/w sugar-water solution with 0.05% of acetone) was deposited in spectrophotometer cuvettes that were placed under the housing beakers (Fig. 1). The initial volume of 40 µL used in OECD bumblebee acute oral guidelines was decreased in this adapted protocol as preliminary tests showed that the wild bees rarely consumed the whole 40 µL droplet before the end of the exposure period. The cuvettes were left in situ for four hours, during which consumption of the solution was visually checked every 30 min (Fig. 1). After visual checking of consumption of the whole 20 μL droplet, the bee was included in the test and marked as a feeder. If the whole solution was not consumed, the bee was marked as non-feeder and discarded from the test. Then, a new capillary containing a 50% w/w untreated sugar-water solution was placed back under the beaker for the remainder of the observation period. To control for evaporation during the exposure period, five additional doses were placed into spectrophotometer cuvettes under empty beakers. These cuvettes were weighed before and after the four-hour exposure period (See Supplementary Fig. S1 for detailed pictures of the protocol).

After the exposure period, sub-lethal effects (e.g., reduced coordination, paralysis, tremors, etc.) were observed and mortality was recorded under red light at 24 h and 48 h after exposure. At 48 h, the final rate of mortality was recorded.

#### Topical exposure

After one day of acclimation, bees were chilled until immobile (max. ten minutes in the freezer at −20 °C, or an hour in the fridge at +4 °C) before handling. They were then exposed by applying a 2 μL droplet of pesticide with a micro-pipette on the dorsal side of the thorax (Fig. 1). The negative control group was treated with 2 μL of distilled water containing 0.05% acetone. To ensure even dispersal of the treatment and control solutions on the bee thorax, Triton® X-100 (0.05%; Fisher Scientific) was used as a surfactant (OECD 2017c). Once the 2 μL droplet was applied, the individual was placed in a Petri dish until it recovered from chilling, then placed back under the inverted see-through plastic beaker of the controlled room with *ad libitum* access to 50% w/w untreated sugar-water solution for the remainder of the observation period (48 h) (See Supplementary Fig. S2 for detailed pictures of the protocol).

During this period, sub-lethal effects were observed and mortality was recorded under red light at 24 and 48 h post-exposure. At 48 h, the final rate of mortality was recorded.

### Statistical analysis

All statistical analyses were carried out in the R environment v 4.1.0 (R Core Team 2023). Generalized linear models (GLM) using package “glmmTMB” v1.1.8 (Brooks et al. 2017) were performed to test the effect of laboratory conditions on the wild bee control groups (1 = dead; 0 = alive) and on the ability to feed on the cuvette (1 = non-feeder; 0 = feeder) as well as to test the effect of the mass on the specific sensitivity. As complete separation occurred in the wild bee mortality data for the sulfoxaflor exposure experiments (Heinze and Schemper 2002), we performed Bayesian GLMs with a Cauchy prior (Gelman et al. 2008) using the function “bayesglm” from the “arm” package v1.13-1 (Gelman and Su 2022). The Kruskal-Wallis rank sum test was performed using the base “stats” package of R to test for differences in the mass of the wild bees. All pairwise tests were performed using package emmeans v1.8.6 (Lenth 2023) and the false discovery rate method to correct the *p*-value for multiple testing.

All statistical omnibus and pairwise statistical tests as well as the generalized linear model were performed using the base stats package of R (R Core Team 2023). Barplots were produced using the ggplot2 package v3.3.5 (Wickham 2016), and mass effect graph was produced using visreg package v2.7.0 (Breheny and Burchett 2020).

## Results

Overall, 1548 females of the 17 targeted wild bee species were sampled and their survival was assessed under laboratory conditions (Table 1). Among the 17 species, nine species were captured in sufficient numbers and survived to the laboratory conditions following our new protocol (Fig. 1). Moreover, we successfully exposed five wild species orally and seven wild species topically (Table 1).

### Mortality and feeding abilities under laboratory conditions

A first attempt following the OECD guidelines for bumblebee testing (OECD 2017b, 2017c) was performed using 7 of the 17 sampled wild bee species (*A. manicatum, N* = *3*; *B. lapidarius, N* = *22*; *C. daviesanus, N* = *3*; *C. hederae, N* = *67*; *D. hirtipes, N* = *3*; *H. signatus, N* = *3*; Table 1). While *B. lapidarius* survived under those conditions, individuals did not feed from the syringe during the exposure period and could not be used in the oral exposure experiment. The flying season of *B. lapidarius* ended before we could use it in a topical exposure experiment. *C. hederae* could not be used because all caught individuals were found to have *Stenoria* larvae attached to them. As the effect of *Stenoria* larval parasitism on the sensitivity to pesticides is not yet known, we decided not to use the species to avoid bias in the results. For the four other species, none of the tested individuals survived the OECD conditions. Therefore, this first attempt led us to use our new protocol described above, i.e., reverted plastic beakers with cotton capillaries during the non-exposure period coupled to spectrophotometry cuvettes during the oral exposure experiment (Fig. 1; Supplementary Figs. S1 and S2).

**Fig. 2:**
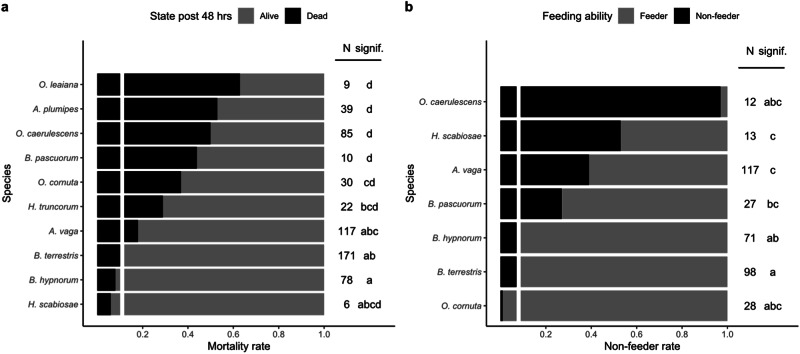
Acclimation and feeding abilities of wild bee species and *B. terrestris*. **a** Mortality rate after 48 h for control individuals under laboratory conditions and **b** non-feeder rate. N = number of tested individuals. Species that do not share the same letter have significantly different proportions at *p* < 0.05 (Bayesian GLMM with binomial family and pairwise comparison using Benjamini–Hochberg correction). The solid white line indicates *B. terrestris* rates of **a** mortality and **b** feeding

The new protocol (one bee per inverted plastic beaker supplied with a soaked cotton capillary) was used for the other 11 wild species and *B. terrestris* (Table 1). First, for *A. fulva*, insufficient individuals (N = 3) were caught (Table 1). However, we noted that all three specimens survived the entire acclimation period and all fed on cuvettes containing a droplet of control solution. On the contrary, while 13 individuals of *C. florisomne* were caught, none of them survived the new protocol. The other nine wild species acclimatized and could be used in the oral (five species) and/or the topical (seven species) exposure experiments (Table 1). Among those nine wild species and *B. terrestris*, the rate of survival of the control groups (i.e., only exposed to the control solution) under laboratory conditions after 48 h differed significantly (Bayesian GLMM with binomial family: χ^2^ = 91.498, df = 9, *p*-value = 8.144e−16). The species that survived significantly better than the others under laboratory conditions and with less than 20% of mortality in the control group were *B. terrestris, H. scabiosae, A. vaga* and *B. hypnorum* (Fig. 2a).

Six out of the nine wild species and *B. terrestris* were exposed to the oral treatment using the cuvette. We found significant differences in their ability to feed on the control solution from the cuvettes (Bayesian GLMM with binomial family, χ^2^ = 117.31, df = 7, *p*-value < 2.2e−16). The species for which most individuals fed on the control solutions were *O. cornuta* and the three *Bombus* species, while none of the *O. caerulescens* individuals fed on the control solution (Fig. 2b). *O. caerulescens* individuals were therefore only used in the topical exposure experiment.

### Effect of oral exposure to Sulfoxaflor

Based on the mortality and feeding results from the control group (Fig. 2), we were able to expose five wild bee species to an oral acute sulfoxaflor dose of 0.563 μg/g body weight. These included two *Bombus* species, *B. hypnorum*, and *B. pascuorum*, as well as *O. cornuta*, *H. scabiosae*, and *A. vaga*. For those species, the treatment, the species as well as the treatment-species interaction factors had significant effects on the probability of mortality (Bayesian GLMM with binomial family; see details in Supplementary Table S6).

**Fig. 3:**
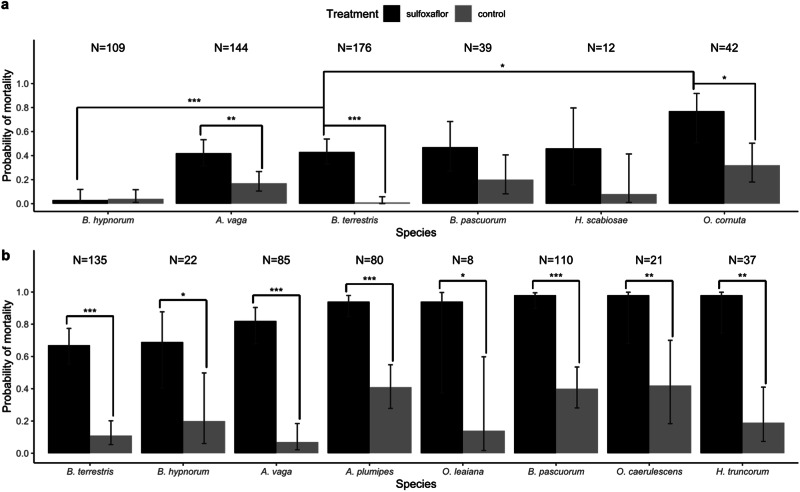
Mortality 48 h after **a** oral ingestion of Sulfoxaflor (0.563 μg/g fresh body weight) and **b** after topical exposure of Sulfoxaflor (10.4 μg/bee). (Bayesian GLMM with binomial family and pairwise comparison using Benjamini–Hochberg correction, Error bars show 95%CI, N = total number of exposed individuals for each species, *p < 0.05, **p < 0.01, ***p < 0.001)

Among the tested bee species, *B. terrestris*, *A. vaga* and *O. cornuta* exhibited sensitivity to acute sulfoxaflor exposure with mortality in treatment group being significantly higher than mortality in their respective control group (Bayesian GLMM with binomial family and pairwise comparison using Benjamini–Hochberg correction: *p*_*B. terrestris*_ = 0.0002, *p*_*A. vaga*_ = 0.0019, *p*_*O. cornuta*_ = 0.0125; Supplementary Tables S7 and S9; Fig. 3a). *B. hypnorum* did not show any significant sensitivity and the two other tested wild bees, *B. pascuorum*, and *H. scabiosae* exhibited non-significant elevated mortality in comparison to the respective control group (Supplementary Tables S7 and S9; Fig. 3a).

Among the species that were sensitive to sulfoxaflor, *O. cornuta* exhibited an elevated probability of mortality in the treatment group with 77.1 ± 0.11% compared to *B. terrestris* with 43.11 ± 0.05% (Bayesian GLMM with binomial family and pairwise comparison using Benjamini–Hochberg correction: *O. cornuta* vs. *B. terrestris* treatment groups *p*-value = 0.0433; Supplementary Tables S7 and S9; Fig. 3a). However, the sensitivity of *A. vaga* towards sulfoxaflor was not significantly different to that of *B. terrestris* (see Supplementary Table S7 for detailed *p*-value information and Supplementary Table S9 for detailed percentages; Fig. 3a).

Among the species that were not significantly affected by sulfoxaflor (*B. hypnorum, B. pascuorum* and *H. scabiosae*), only *B. hypnorum* could be considered as less sensitive than *B. terrestris* given that there was no significant difference between the control and the treatment group in terms of mortality with large sample size and that the sulfoxaflor treatment group showed, therefore, a significantly lower mortality than *B. terrestris* (Bayesian GLMM with binomial family and pairwise comparison using Benjamini–Hochberg correction: *B. hypnorum* vs. *B. terrestris* treatment groups *p*-value = 0.0005; Supplementary Table S7 and S9, Fig. 3a). However, more replicates would be necessary to determine the difference in sensitivity for the other species, i.e., *B. pascuorum* (N = 39), *and H. scabiosae* (N = 12).

### Effect of topical exposure to Sulfoxaflor

In addition to the reference species *B. terrestris*, seven wild bee species were topically exposed to an acute sulfoxaflor dose of 10.4 μg/bee, namely *B. hypnorum* and *B. pascuorum*, *H. truncorum*, *O. leaiana* and *O. caerulescens*, as well as *A. vaga* and *H. scabiosae*. Only the species and the treatment, but not the species-treatment interaction had a significant effect on the probability of mortality in the tested wild bees (Bayesian GLMM with binomial family, Supplementary Table S6).

A significantly elevated mortality could be observed in all tested species after acute sulfoxaflor exposure (Fig. 3b; see Supplementary Table S8 for detailed *p*-value information and Supplementary Table S9 for detailed percentages). However, in contrast to the oral exposure experiment, the species-treatment interaction factor had no significant effect on the probability of mortality. No difference in terms of topical sensitivity could be highlighted between the species and therefore, no species more sensitive than *B. terrestris* was found in this experiment. However, it is important to note that more replicates would be needed to conclude on the topical sensitivity of *B. hypnorum*, *O. leaiana* and *O. caerulescens*.

### Relation between fresh weight and rate of mortality

**Fig. 4:**
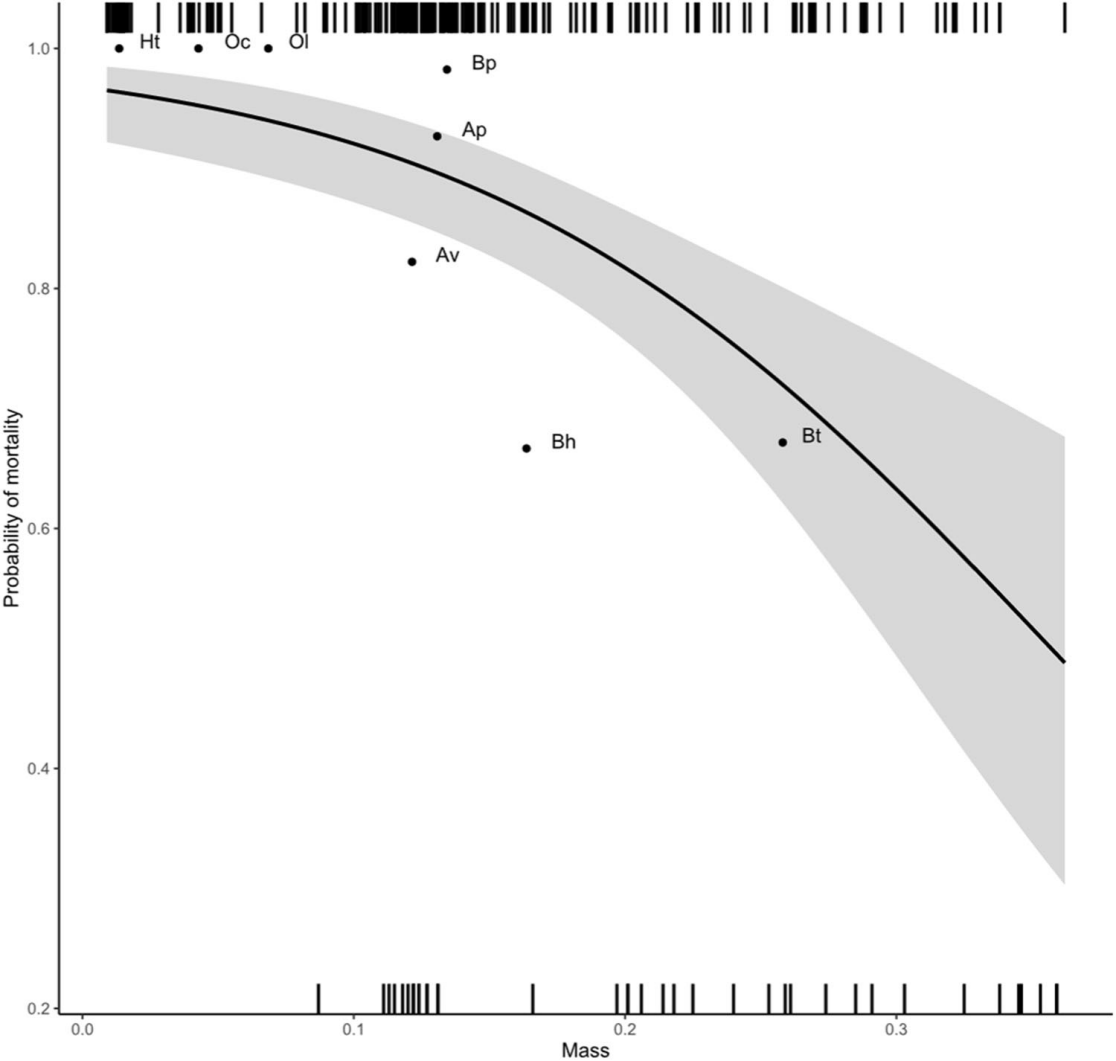
Effect of fresh weight on the sensitivity towards topical exposure of sulfoxaflor (10.4 μg/bee; GLMM with family binomial: *p* = 6.798e^−06^). The dots indicate the mean specific weight and sensitivity of the species while dashes on the abscissa locate the survival (upper dashes, dead; lower dashes, alive). Ht *H. truncorum*, Oc *O. caerulescens*, Ol *O. leaiana*, Bp *B. pascuorum*, Ap *A. plumipes*, Av *A. vaga*, Bh *B. hypnorum*, and Bt *B. terrestris*

Experimental topical exposure was conducted on eight bee species varying considerably in fresh weight, with the lightest species, *H. truncorum*, being on average almost twenty times lighter than the largest one, *B. terrestris* (pairwise comparison using Wilcoxon rank sum test: m_Ht_ = 0.014 g and m_Bt_ = 0.261 g; *p*-value < 2e−16). It appeared that weight is negatively related to mortality across species, with the heaviest individuals being significantly less sensitive than the lightest (GLMM with family binomial: χ² = 20.25, df. = 1, *p*-value = 6.798e^−06^). It should be noted that, in the three smallest species, every treated individual died after 48 h (Fig. 4).

## Discussion

### Mortality and feeding abilities under laboratory conditions

We tested 17 wild bee species in a controlled housing and feeding assay, of which 11 acclimatized and were employed in our adjusted protocol for ecotoxicological testing of wild bee species (Fig. 1). Nine of the 11 survived for at least 48 h post-collection, and five were able to feed on a solution from a cuvette, in a number sufficient to compare their sensitivity to the insecticide sulfoxaflor.

Maintenance in captivity can enhance survival but can be also very stressful for wild animals (Mason 2010). The evidence of compromised well-being of wild animals often suggests that physiological or psychological needs are not being met when held captive (e.g. Barnes et al. 2002). There is little information on the impact of captivity on insects in general and wild bees in particular. In our study, many solitary bee species had difficulties or failed to feed on sirup dispensers, making oral exposure experiments impossible to perform. Recent studies of wild bees showed enhanced survival rates and feeding success using a “group feeding” method (Phan et al. 2020; Sampson et al. 2023) and dental wicks as feeders by 12 American wild bee species (Sampson et al. 2023) or artificial flowers by one Brazilian species (Tadei et al. 2019). As of today, there are no guidelines for standardized acute exposure and lower-tier hazard assessments for solitary bees, mainly due to the lack of standardized feeding methods and the difficulty of maintaining them under lab conditions (EFSA et al. 2023). These alternative methods, and especially the petal feeding method proposed for *Osmia* spp. (Azpiazu et al. 2023), should be further developed in future experiments to develop new standardized protocols for solitary bees.

The level of stress generated by captivity is species-specific (Pearce-Kelly et al. 2007). This could be due for example to a high metabolic rate probably species-specific, notably for *A. plumipes*, as it was demonstrated for another species of the same tribe, *Amegilla chlorocyanea* (Tomlinson et al. 2015). Increasing the space to allow free flying and adequate lighting and temperature might be additional potential solutions to explore to enhance the acceptance by wild bees of being kept in captivity (Leonard and Harmon-Threatt 2019). In addition to the known conditions for *A. mellifera* and *Bombus* spp rearing (OECD 2017a; Tasei and Aupinel 2008), the temperature and lighting parameters have been explored for some wild bees especially from the Halictini tribe (Bell 1973; Greenberg 1982; Kamm 1974; Plateaux-Quénu 1992) and the Osmia genus (Eeraerts et al. 2020; Hellström et al. 2023; Medrzyck et al. 2021).

The optimal temperature and lighting conditions may vary between genera or even species. Using a protocol such as that developed in our study, it could be possible to collect new maintenance data on other species and to allow the grouping of several species with similar optimal conditions to establish condition-specific protocols enhancing the survivability of diverse species at the same time in relation to their traits (e.g. size).

### Sensitivity of wild bees to oral and topical sulfoxaflor exposure

Using our new protocol, when compared to the reference species (*B. terrestris*), we found (i) more sensitive species: *O. cornuta* (oral); (ii) less sensitive species: *B. hypnorum* (oral), as well as tendencies to vary from *B. terrestris* in sensitivity after topical exposure for *A. plumipes*, *B. pascuorum O. cornuta* (topical), and *H. truncorum*. Our results confirm therefore previous results of Arena and Sgolastra’s (2014) meta-analysis showing a wide variability in terms of sensitivity amongst bee species.

Wild bees exhibit species-specific sensitivity towards acute pesticide exposure, and they can exhibit a higher sensitivity than the reference species currently used (i.e. *A. mellifera* and *B. terrestris*). However, our results need to be interpreted with caution. Indeed, for some species such as *H. scabiosae*, *H. truncorum*, *O. caerulescens* or *O. leaiana*, sample sizes were much lower than those recommended by the OECD guidelines (OECD 2017b, 2017c). We should therefore recommend further studies repeated with an increased sample size.

Our study suggests that differences in sensitivity can be partly explained by an individual’s fresh weight, with heavier individuals being less sensitive than lighter ones (Fig. 4). This result was expected as there is a decrease in the surface area-to-volume ratio when size increases (Devillers et al. 2003; Johansen 1972). At the intra-specific level, Arena and Sgolastra (2014) already found a negative correlation between individual body weight and sensitivity towards pesticides. As discussed in Pamminger (2021), body weight is an important predictor of the sensitivity of wild bees and it should therefore be systematically considered in the risk assessment protocols. As all species received the same amount of sulfoxaflor in this study, results for small species should be treated with caution. In this context, those species are overexposed, and comparison with other species would be misleading. The results of this study concerning small species should only be used to determine which species could be used in future work. For further studies using such species, doses should be adapted to their weight.

However, as suggested by Linguadoca et al. (2022) the variability in terms of pesticide sensitivity found amongst bee species cannot be explained by a single morphological trait. Indeed, several physiological and morphological traits are known to influence in different ways the sensitivity of a bee to pesticides (Arena and Sgolastra 2014; Uhl et al. 2016). First, sensitivity may vary according to external physiological traits such as hairiness as well as cuticle composition and thickness as these characteristics drive the rate at which insecticide enters the insect’s body (Balabanidou et al. 2018; Lewis 1980). Regarding internal traits, the sensitivity of an individual or a species to pesticides is also directly related to its mechanisms of detoxification (Beadle et al. 2019; Roush and Tabashnik 1990). In some cases, sensitivity seems more related to detoxification efficiency than body weight, especially with neonicotinoids (Pamminger 2021). Managed bee species have specific P450 enzymes that are preadapted to confer intrinsic tolerance to some insecticides. In contrast, some wild species, i.e. species from the Megachilini, Lithurgini and Anthidini tribes (Megachilidae family), lack CYP9Q-related P450s and have been shown to be more sensitive to neonicotinoid thiacloprid and imidacloprid insecticides (Hayward et al. 2024, 2019). However, it has been hypothesized that these deficits in detoxification genes would increase bee sensitivity to interactions among toxicants (Berenbaum and Johnson 2015), which could partly explain our results with a single molecule exposure. Some species have the physiological ability to detoxify phytochemicals, such as alkaloids contained in the floral resources of some plant species (Cresswell et al. 2012; Elliott et al. 2008). These abilities could help in the detoxification of pesticides, such as neonicotinoids (Cresswell et al. 2012). For example, *H. truncorum*, and *O. leaiana* are oligolectic on the Asteraceae plant family, members of which have been shown to contain toxic secondary compounds in their pollen and nectar (Vanderplanck et al. 2020). We did not find a significant difference in their sensitivity to pesticides compared to *B. terrestris*, possibly due to the low number of tested specimens. It would therefore be interesting to continue experimenting on these species and other oligolectic species to explore mechanisms explaining the species-specific variation in sensitivity to insecticides. Broadly speaking, generalist species are better represented in ecotoxicological studies than specialists and new models have been suggested to address this gap as with *Osmia brevicornis*, a pollen specialist on plants from the Brassicaceae family (Hellström et al. 2023). Exposure probability between polylectic and oligolectic species can also be questioned. We can expect polylectic species to be generally more exposed by feeding on various crops, and oligolectic species to be generally less exposed by feeding on non-cultivated plants. However, various oligolectic species can be found nesting or foraging on plants near crop edges (e.g. *C. hederae* on ivy or *A. vaga* on willow), which will also lead to exposition, or can even forage on crop plants, which can be the case for *H. scabiosae* and *H. truncorum* for example that mainly feed on Asteraceae plants such as *Helianthus sp*., and O. brevicornis that mainly feed on Brassicacea plants such as oil seed rape (Hellström et al. 2023).

Finally, detoxification processes are controlled by enzymatic activity, which may vary from one species to another due to, among other parameters, differences in hemolymph pH (Uhl et al. 2016). For example, the LD30 (i.e. dose at which 30 per cent of the sample died) measured for trichlorfon in *A. mellifera*, and *Megachile rotundata* were respectively 28.5 µg/g b.w. and 515.0 µg/g, while the respective hemolymph pH measured at 6.0, and 6.8. It has been hypothesized that xenobiotics can be detoxified at different rates depending on the species in relation to the specific pH of its hemolymph (Ahmad and Johansen 1973; Haas et al. 2022; Hayward et al. 2019). Analyzing the hemolymph of species with a different insecticide sensitivity to *B. terrestris* could provide more insight into the underlying mechanisms of detoxification efficiency and its variation in relation to sensitivity.

### Perspectives

Wild bees are difficult to maintain in captivity. To compensate for their high intrinsic lab mortality, a large number of individuals need to be taken from the wild, which is not always possible for small bee communities, and for bees that do not nest in aggregations. Moreover, the high percentage of non-feeders found during our oral exposure experiment, and the high rates of mortality found in control groups, further diminished sample sizes; laboratory conditions must be improved to allow ecotoxicological testing of a larger spectrum of wild bee species. For further experiments, and to increase the sample size, some methods that have been developed to adapt the feeding of bees to their natural feeding behavior can be used. For example, a flower from which the reproductive column was removed and replaced by the test solution has already been used with *Megachile rotundata* and *Osmia lignaria* (Ladurner et al. 2003). While difficult to install and to adapt to specialist bees, this technique has been improved by the “petal method” which seems to increase feeding success and to be easily set up. This method consists of using a single petal as a visual clue to indicate the treatment solution and facilitate feeding by solitary bees (Hellström et al. 2023; Hinarejos et al. 2015).

Another method has been recently developed (Kueh Tai et al. 2022), using pins crossed over the gaster to restrain *Leioproctus paahaumaa* individuals and feeding them with 10 µL of an insecticide-sucrose solution. Individuals were then transferred into plastic jars in groups of 10 and fed *ad libitum* through a cotton wick soaked with 50% w/v sucrose solution. This kind of group feeding method could be less stressful, especially for gregariously nesting wild bee species, and could reduce the mortality observed in our study.

To increase survival under laboratory conditions, the stress caused by having been caught in the wild could also be avoided by rearing the species under laboratory conditions, from the first larval stages until emergence (Eeraerts et al. 2020). However, these methods are only known for a few species (Claus et al. 2021; Peterson and Artz 2014), particularly for ground-nesting species (e.g. Plateaux-Quénu et al. 2000) which represent the majority of bee diversity (Michener 2007).

Finally, while our results show that many species do not survive well in the laboratory, they also show that these methods can work on some species with interesting ecological traits (i.e. *A. plumipes* as a soil-nesting solitary bee, or *A. vaga* as an oligolectic bee species) that are not shared with the main model species. A big gap in knowledge about the pesticide’s lethal and sub-lethal effects on bees remains to be filled. Moreover, as highlighted in the recent revised guidance document of the EFSA et al. 2023, the use of models allowing extrapolation of the sensitivity of wild bees towards pesticides is likely to play a key role in future risk assessments. As for now, the number of available mortality data is not sufficient to predict with any certainty the variation in sensitivity that may occur among wild bees, especially regarding oligolectic species (Hellström et al. 2023). More importantly, genetic and molecular data needs to be collected to fully comprehend the mechanisms underlying the variation between species and use it in models confidently extrapolating the sensitivity of wild bees towards a new molecule (EFSA et al. 2023).

While the diversity of plant protection products is increasing due to the emergence of pest resistance, their toxic effects are still mainly studied on a single bee species, *A. mellifera*. Fortunately, risk assessments have recently begun to include non-*Apis* species, such as *B. terrestris* and *Osmia bicornis*. However, the high ecological, morphological and physiological variability found amongst the 20,000 bee species urges the development of adapted methodologies for their breeding, exposure to pesticides and determination of the characteristics that cause the variation in sensitivity.

### Supplementary information

Supplementary Information

## References

[CR1] Ahmad Z, Johansen C (1973). Selective Toxicity of Carbophenothion and Trichlorfon to the Honey Bee and the Alfalfa Leafcutting Bee. Environ Entomol.

[CR2] Arena M, Sgolastra F (2014). A meta-analysis comparing the sensitivity of bees to pesticides. Ecotoxicology.

[CR3] Azpiazu C, Hinarejos S, Sancho G, Albacete S, Sgolastra F, Martins CAH, Domene X, Benrezkallah J, Rodrigo A, Arnan X, Bosch J (2023). Description and validation of an improved method to feed solitary bees (Osmia spp.) known amounts of pesticides. Ecotoxicol Environ Saf.

[CR4] Balabanidou V, Grigoraki L, Vontas J (2018). Insect cuticle: a critical determinant of insecticide resistance. Curr Opin Insect Sci.

[CR5] Barnes R, Greene K, Holland J, Lamm M (2002). Management and husbandry of duikers at the Los Angeles Zoo. Zoo Biol.

[CR6] Beadle K, Singh KS, Troczka BJ, Randall E, Zaworra M, Zimmer CT, Hayward A, Reid R, Kor L, Kohler M, Buer B, Nelson DR, Williamson MS, Davies TGE, Field LM, Nauen R, Bass C (2019). Genomic insights into neonicotinoid sensitivity in the solitary bee *Osmia bicornis*. PLoS Genet.

[CR7] Bell WJ (1973). Factors controlling initiation of vitellogenesis in a primitively social bee, *Lasioglossum zephyrum* (Hymenoptera: Halictidae). Ins Soc.

[CR8] Berenbaum MR, Johnson RM (2015). Xenobiotic detoxification pathways in honey bees. Curr Opin Insect Sci.

[CR9] Breheny P, Burchett W (2020). Visualization of Regression Models Using visreg.. The R J.

[CR10] Brooks ME, Kristensen K, Van Benthem KJ, Magnusson A, Berg CW, Nielsen A, Skaug HJ, Mächler M, Bolker BM (2017) Modeling zero-inflated count data with glmmTMB. Ecology. 10.1101/132753

[CR11] Buchmann SL, Nabhan GP (1997) The forgotten pollinators, 1st ed. Island Press, Washington DC, USA

[CR12] Cameron SA, Lozier JD, Strange JP, Koch JB, Cordes N, Solter LF, Griswold TL (2011). Patterns of widespread decline in North American bumble bees. Proc Natl Acad Sci.

[CR13] Claus G, Pisman M, Spanoghe P, Smagghe G, Eeraerts M (2021). Larval oral exposure to thiacloprid: Dose-response toxicity testing in solitary bees, Osmia spp. (Hymenoptera: Megachilidae). Ecotoxicol Environm Saf.

[CR14] Cresswell JE, Page CJ, Uygun MB, Holmbergh M, Li Y, Wheeler JG, Laycock I, Pook CJ, de Ibarra NH, Smirnoff N, Tyler CR (2012). Differential sensitivity of honey bees and bumble bees to a dietary insecticide (imidacloprid). Zoology.

[CR15] Danforth BN, Cardinal S, Praz C, Almeida EAB, Michez D (2013). The Impact of Molecular Data on Our Understanding of Bee Phylogeny and Evolution. Annu Rev Entomol.

[CR16] Devillers J, Decourtye A, Budzinski H, Pham-Delègue MH, Cluzeau S, Maurin G (2003). Comparative toxicity and hazards of pesticides to *Apis* and non- *Apis* bees. A chemometrical study. SAR QSAR Environ Res.

[CR17] Dicks LV, Breeze TD, Ngo HT, Senapathi D, An J, Aizen MA, Basu P, Buchori D, Galetto L, Garibaldi LA, Gemmill-Herren B, Howlett BG, Imperatriz-Fonseca VL, Johnson SD, Kovács-Hostyánszki A, Kwon YJ, Lattorff HMG, Lungharwo T, Seymour CL, Vanbergen AJ, Potts SG (2021). A global-scale expert assessment of drivers and risks associated with pollinator decline. Nat Ecol Evol.

[CR18] Drossart M, Rasmont P, Vanormelingen P, Dufrêne M, Folschweiller M, Pauly A, Vereecken NJ, Vray S, Zambra E, D’Haeseleer J, Michez D (2019) Belgian Red List of Bees, Belgian Science Policy 2018 (BRAIN-be - (Belgian Research Action through Interdisciplinary Networks). Presse universitaire de l’Université de Mons, Mons

[CR19] Duchenne F, Thébault E, Michez D, Gérard M, Devaux C, Rasmont P, Vereecken NJ, Fontaine C (2020). Long‐term effects of global change on occupancy and flight period of wild bees in Belgium. Glob Change Biol.

[CR20] Eeraerts M, Pisman M, Vanderhaegen R, Meeus I, Smagghe G (2020). Recommendations for standardized oral toxicity test protocols for larvae of solitary bees, Osmia spp. Apidologie.

[CR21] EFSA, Adriaanse P, Arce A, Focks A, Ingels B, Jölli D, Lambin S, Rundlöf M, Süßenbach D, Del Aguila M, Ercolano V, Ferilli F, Ippolito A, Szentes C, Neri FM, Padovani L, Rortais A, Wassenberg J, Auteri D (2023) Revised guidance on the risk assessment of plant protection products on bees (*Apis mellifera*, *Bombus* spp. and solitary bees). EFS2 21, 10.2903/j.efsa.2023.7989 10.2903/j.efsa.2023.7989 PMC10173852 37179655

[CR22] Elliott SE, Irwin RE, Adler LS, Williams NM (2008). The nectar alkaloid, gelsemine, does not affect offspring performance of a native solitary bee, *Osmia lignaria* (Megachilidae). Ecol Entomol.

[CR23] European Food Safety Authority (2014) Conclusion on the peer review of the pesticide risk assessment of the active substance sulfoxaflor. EFS2 12. 10.2903/j.efsa.2014.3692 10.2903/j.efsa.2014.3692 PMC13159879 42125401

[CR24] European Food Safety Authority (EFSA) (2013) Guidance on the risk assessment of plant protection products on bees (*Apis mellifera*, *Bombus* spp. and solitary bees). EFS2 11. 10.2903/j.efsa.2013.3295 10.2903/j.efsa.2023.7989 PMC10173852 37179655

[CR25] European Food Safety Authority (EFSA), Anastassiadou M, Arena M, Auteri D, Brancato A, Bura L, Carrasco Cabrera L, Chaideftou E, Chiusolo A, Court Marques D, Crivellente F, De Lentdecker C, Egsmose M, Fait G, Greco L, Ippolito A, Istace F, Jarrah S, Kardassi D, Leuschner R, Lostia A, Lythgo C, Magrans O, Mangas I, Miron I, Molnar T, Padovani L, Parra Morte JM, Pedersen R, Reich H, Santos M, Serafimova R, Sharp R, Stanek A, Sturma J, Szentes C, Terron A, Tiramani M, Vagenende B, Villamar‐Bouza L (2020) Peer review of the pesticide risk assessment for the active substance sulfoxaflor in light of confirmatory data submitted. EFS2 18, 10.2903/j.efsa.2020.6056

[CR26] Fiordaliso W, Reverté S, Wood T, Barbier Y, Rasmont P, Lefèbvre A, Loockx M, Reese A, Ruelle E, Michez D (2022). Inventaire et conservation des abeilles sauvages (Hymenoptera: Anthophila) du sillon industriel hainuyer (Belgique). Belgian J Entomol.

[CR27] Franklin EL, Raine NE (2019). Moving beyond honeybee-centric pesticide risk assessments to protect all pollinators. Nat Ecol Evol.

[CR28] Gelman A, Jakulin A, Pittau MG, Su Y-S (2008) A weakly informative default prior distribution for logistic and other regression models. Ann Appl Stat 2, 10.1214/08-AOAS191

[CR29] Gelman A, Su Y-S (2022) arm: Data Analysis Using Regression and Multilevel/Hierarchical Models. R package version 1.13-1, https://CRAN.R-project.org/package=arm

[CR30] Ghisbain G (2021). Are Bumblebees Relevant Models for Understanding Wild Bee Decline?. Front. Conserv. Sci..

[CR31] Ghisbain G, Rosa P, Bogush P, Flaminio S, Le Divelec R, Dorchin A, Kasparek M, Kuhlmann M, Litman J, Mignot M, Müller A, Praz C, Radchenko VG, Rasmont P, Risch S, Robert SPM, Smit J, Wood TJ, Michez D, Reverté S (2023). The new annotated checklist of the wild bees of Europe (Hymenoptera: Anthophila). Zootaxa.

[CR32] Godfray HCJ, Blacquière T, Field LM, Hails RS, Petrokofsky G, Potts SG, Raine NE, Vanbergen AJ, McLean AR (2014). A restatement of the natural science evidence base concerning neonicotinoid insecticides and insect pollinators. Proc R Soc B.

[CR33] Goulson D, Nicholls E, Botias C, Rotheray EL (2015). Bee declines driven by combined stress from parasites, pesticides, and lack of flowers. Science.

[CR34] Greenberg L (1982). Year-Round Culturing and Productivity of a Sweat Bee, *Lasioglossum zephyrum* (Hymenoptera: Halictidae). J Kansas Entomol Soc.

[CR35] Haas J, Hayward A, Buer B, Maiwald F, Nebelsiek B, Glaubitz J, Bass C, Nauen R (2022). Phylogenomic and functional characterization of an evolutionary conserved cytochrome P450-based insecticide detoxification mechanism in bees. Proc Natl Acad Sci USA.

[CR36] Hayward A, Beadle K, Singh KS, Exeler N, Zaworra M, Almanza M-T, Nikolakis A, Garside C, Glaubitz J, Bass C, Nauen R (2019). The leafcutter bee, *Megachile rotundata*, is more sensitive to N-cyanoamidine neonicotinoid and butenolide insecticides than other managed bees. Nat Ecol Evol.

[CR37] Hayward A, Hunt BJ, Haas J, Bushnell‐Crowther E, Troczka BJ, Pym A, Beadle K, Field J, Nelson DR, Nauen R, Bass C (2024). A cytochrome P450 insecticide detoxification mechanism is not conserved across the Megachilidae family of bees. Evol Appl.

[CR38] Heinze G, Schemper M (2002). A solution to the problem of separation in logistic regression. Statist Med.

[CR39] Hellström S, Strobl V, Straub L, Osterman WHA, Paxton RJ, Osterman J (2023). Beyond generalists: The Brassicaceae pollen specialist *Osmia brevicornis* as a prospective model organism when exploring pesticide risk to bees. Environ Sustain Indic.

[CR40] Helson BV, Barber KN, Kingsbury PD (1994) Laboratory toxicology of six forestry insecticides to four species of bee (hymenoptera: Apoidea). Arch Environ Contam Toxicol 27, 10.1007/BF00203895

[CR41] Hinarejos S, Domene X, Bosch J (2015) Oral toxicity of dimethoate to adult *Osmia cornuta* using an improved laboratory feeding method for solitary bees. In: 12th Int. Symp. of ICP-PR Hazards of Pesticides to Bees, Ghent, Belgium, 15–17 September 2014. Julius-Kuhn-Arch. 450:192

[CR42] Johansen CA (1972). Toxicity of Field-Weathered Insecticide Residues to Four Kinds of Bees. Environ Entomol.

[CR43] Johnson R, Corn ML (2015). Bee Health: The Role of Pesticides, in: Bee Health: Background, Issues and the Role of Pesticides, Insects and Other Terrestrial Arthropods: Biology, Chemistry and Behavior.

[CR44] Kamm DR (1974). Effects of Temperature, Day Length, and Number of Adults on the Sizes of Cells and Offspring in a Primitively Social Bee (Hymenoptera: Halictidae). J Kansas Entomol Soc.

[CR45] Krupke CH, Hunt GJ, Eitzer BD, Andino G, Given K (2012). Multiple Routes of Pesticide Exposure for Honey Bees Living Near Agricultural Fields. PLoS One.

[CR46] Kueh Tai F, Pattemore DE, Jochym M, Beggs JR, Northcott GL, Mortensen AN (2022). Honey bee toxicological responses do not accurately predict environmental risk of imidacloprid to a solitary ground-nesting bee species. Sci Total Environ.

[CR47] Ladurner E, Bosch J, Maini S, Kemp WP (2003). A method to feed individual bees (Hymenoptera: Apiformes) known amounts of pesticides. Apidologie.

[CR48] Lenth RV (2023) emmeans: Estimated Marginal Means, aka Least-Squares Means. R package version 1.8.6, https://CRAN.R-project.org/package=emmeans

[CR49] Leonard RJ, Harmon-Threatt AN (2019). Methods for rearing ground-nesting bees under laboratory conditions. Apidologie.

[CR50] Lewis CT (1980) The Penetration of Cuticle by Insecticides, in: Miller TA (ed), Cuticle Techniques in Arthropods, Springer Series in Experimental Entomology. Springer New York, New York, NY, pp. 367–400. 10.1007/978-1-4612-6076-9_10

[CR51] Linguadoca A, Jürison M, Hellström S, Straw EA, Šima P, Karise R, Costa C, Serra G, Colombo R, Paxton RJ, Mänd M, Brown MJF (2022). Intra-specific variation in sensitivity of *Bombus terrestris* and *Osmia bicornis* to three pesticides. Sci Rep.

[CR52] Mason GJ (2010). Species differences in responses to captivity: stress, welfare and the comparative method. Trends Ecol Evol.

[CR53] Medrzyck P, Hellström S, Straw E, Linguadoca A, Jürison M, Alaux C, Barascou L, Brown MJF, Costa C, De la Rúa P, de Miranda JR, di Prisco G, Forsgren E, Karise R, Le-Conte Y, Mänd M, Martínez-López V, Neumann P, Onorati P, Paxton RJ, Sene D, Strobi V, Yanez O (2021) Improved protocols for testing agrochemicals in bees. (Deliverable D No. 3). EU Horizon 2020 PoshBee Project, Grant agreement No. 773921.

[CR54] Michener CD (2007). The bees of the world, 2nd ed.

[CR55] Michez D, Rasmont P, Terzo M, Vereecken NJ (2019). Bees of Europe, 1st ed.

[CR56] Nieto A, Roberts SPM, Kemp J, Rasmont P, Kuhlmann M, Criado MG, Biesmeijer JC, Bogush P, Dathe HH, De la Rúa P, De Meulemeester T, Dehon M, Dewulf A, Ortiz-Sánchez FJ, Lhomme P, Pauly A, Potts SG, Praz C, Quaranta M, Radchenko VG, Scheuchl E, Smit J, Straka J, Terzo M, Tomozii B, Window J, Michez D (2014). European red list of bees.

[CR57] OECD (2017a) Test No. 245: Honey Bee (*Apis Mellifera* L.), Chronic Oral Toxicity Test (10-Day Feeding), OECD Guidelines for the Testing of Chemicals, Section 2. OECD, 10.1787/9789264284081-en

[CR58] OECD (2017b) Test No. 246: Bumblebee, Acute Contact Toxicity Test, OECD Guidelines for the Testing of Chemicals, Section 2. OECD, 10.1787/9789264284104-en

[CR59] OECD (2017c) Test No. 247: Bumblebee, Acute Oral Toxicity Test, OECD Guidelines for the Testing of Chemicals, Section 2. OECD, 10.1787/9789264284128-en

[CR60] OECD (1998a) Test No. 214: Honeybees, Acute Contact Toxicity Test, OECD Guidelines for the Testing of Chemicals, Section 2. OECD, 10.1787/9789264070189-en

[CR61] OECD (1998b) Test No. 213: Honeybees, Acute Oral Toxicity Test, OECD Guidelines for the Testing of Chemicals, Section 2. OECD, 10.1787/9789264070165-en

[CR62] Ollerton J, Winfree R, Tarrant S (2011). How many flowering plants are pollinated by animals?. Oikos.

[CR63] Pamminger T (2021). Extrapolating Acute Contact Bee Sensitivity to Insecticides Based on Body Weight Using a Phylogenetically Informed Interspecies Scaling Framework. Environ Toxicol Chem.

[CR64] Pearce-Kelly P, Morgan R, Honan P, Barrett P, Perrotti L, Magdich M, Spencer W (2007). The conservation value of insect breeding programmes: rationale, evaluation tools and example programme case studies, in: Insect Conservation Biology.

[CR65] Persson AS, Rundlöf M, Clough Y, Smith HG (2015). Bumble bees show trait-dependent vulnerability to landscape simplification. Biodivers Conserv.

[CR66] Peterson, SS, Artz, DR, 2014. Production of Solitary Bees for Pollination in the United States, in: Mass Production of Beneficial Organisms. Elsevier, 653–681. 10.1016/B978-0-12-391453-8.00019-4

[CR67] Phan NT, Joshi NK, Rajotte EG, López-Uribe MM, Zhu F, Biddinger DJ (2020). A new ingestion bioassay protocol for assessing pesticide toxicity to the adult Japanese orchard bee (*Osmia cornifrons*). Sci Rep.

[CR68] Plateaux-Quénu C (1992). Comparative biological data in two closely related eusocial species: *Evylaeus calceatus* (Scop.) and *Evylaeus albipes* (F.) (Hym., Halictinae). Ins Soc.

[CR69] Plateaux-Quénu C, Plateaux L, Packer L (2000). Population-typical behaviours are retained when eusocial and non-eusocial forms of *Evylaeus albipes* (F.) (Hymenoptera, Halictidae) are reared simultaneously in the laboratory. Insectes Soc.

[CR70] Prado A, Requier F, Crauser D, Le Conte Y, Bretagnolle V, Alaux C (2020). Honeybee lifespan: the critical role of pre-foraging stage. R Soc Open Sci.

[CR93] Przybyla K, Michez D, Rasmont P, Habay J (2023). Notes sur la nidification spontanée de reines sauvages de bourdons en Belgique (Hymenoptera : Apidae).. Osmia.

[CR72] R Core Team (2023) R: A Language and Environment for Statistical Computing. R Foundation for Statistical Computing, Vienna, Austria. https://www.R-project.org/

[CR73] Rasmont P, Coppee A, Michez D, De Meulemeester T (2008). An overview of the *Bombus terrestris* (L. 1758) subspecies (Hymenoptera: Apidae). Int J Entomol.

[CR74] Roush RT, Tabashnik BE (1990). Pesticide Resistance in Arthropods.

[CR75] Rundlöf M, Andersson GKS, Bommarco R, Fries I, Hederström V, Herbertsson L, Jonsson O, Klatt BK, Pedersen TR, Yourstone J, Smith HG (2015). Seed coating with a neonicotinoid insecticide negatively affects wild bees. Nature.

[CR76] Sampson B, Gregorc A, Alburaki M, Werle C, Karim S, Adamczyk J, Knight P (2023). Sensitivity to imidacloprid insecticide varies among some social and solitary bee species of agricultural value. PLoS ONE.

[CR77] Scott-Dupree CD, Conroy L, Harris CR (2009). Impact of Currently Used or Potentially Useful Insecticides for Canola Agroecosystems on *Bombus impatiens* (Hymenoptera: Apidae), *Megachile rotundata* (Hymenoptera: Megachilidae), and *Osmia lignaria* (Hymenoptera: Megachilidae). J Econ Entomol.

[CR78] Sgolastra F, Hinarejos S, Pitts-Singer TL, Boyle NK, Joseph T, Lūckmann J, Raine NE, Singh R, Williams NM, Bosch J (2019). Pesticide Exposure Assessment Paradigm for Solitary Bees. Environ Entomol.

[CR79] Spurgeon D, Hesketh H, Lahive E, Svendsen C, Baas J, Robinson A, Horton A, Heard M (2016) Chronic oral lethal and sub‐lethal toxicities of different binary mixtures of pesticides and contaminants in bees (*Apis mellifera*, *Osmia bicornis* and *Bombus terrestris*). EFS3 13, 10.2903/sp.efsa.2016.EN-1076

[CR80] Straka J, Černá K, Macháčková L, Zemenová M, Keil P (2014). Life span in the wild: the role of activity and climate in natural populations of bees. Funct Ecol.

[CR81] Tadei R, Domingues CEC, Malaquias JB, Camilo EV, Malaspina O, Silva-Zacarin ECM (2019). Late effect of larval co-exposure to the insecticide clothianidin and fungicide pyraclostrobin in Africanized *Apis mellifera*. Sci Rep.

[CR82] Tadei R, Silva CI, Decio P, Silva‐Zacarin ECM, Malaspina O (2022). Method for maintaining adult solitary bee *Centris analis* under laboratory conditions. Methods Ecol Evol.

[CR83] Tasei J-N, Aupinel P (2008). Validation of a Method Using Queenless <I>Bombus terrestris</I> Micro-Colonies for Testing the Nutritive Value of Commercial Pollen Mixes by Comparison with Queenright Colonies. J Econ Entomol.

[CR84] Thompson HM (2001). Assessing the exposure and toxicity of pesticides to bumblebees (sp.). Apidologie.

[CR85] Tomlinson S, Dixon KW, Didham RK, Bradshaw SD (2015). Physiological plasticity of metabolic rates in the invasive honey bee and an endemic Australian bee species. J Comp Physiol B.

[CR86] Uhl P, Franke LA, Rehberg C, Wollmann C, Stahlschmidt P, Jeker L, Brühl CA (2016). Interspecific sensitivity of bees towards dimethoate and implications for environmental risk assessment. Sci Rep.

[CR87] Vanderplanck M, Gilles H, Nonclercq D, Duez P, Gerbaux P (2020). Asteraceae Paradox: Chemical and Mechanical Protection of Taraxacum Pollen. Insects.

[CR88] Vray S, Rollin O, Rasmont P, Dufrêne M, Michez D, Dendoncker N (2019). A century of local changes in bumblebee communities and landscape composition in Belgium. J Insect Conserv.

[CR89] Wickham H (2016). ggplot2: Elegant Graphics for Data Analysis, 2nd ed, Use R!.

[CR90] Williams GR, Alaux C, Costa C, Csáki T, Doublet V, Eisenhardt D, Fries I, Kuhn R, McMahon DP, Medrzycki P, Murray TE, Natsopoulou ME, Neumann P, Oliver R, Paxton RJ, Pernal SF, Shutler D, Tanner G, van der Steen JJM, Brodschneider R (2013). Standard methods for maintaining adult *Apis mellifera* in cages under in vitro laboratory conditions. J Apicultural Res.

[CR91] Wood TJ, Michez D, Paxton RJ, Drossart M, Neumann P, Gérard M, Vanderplanck M, Barraud A, Martinet B, Leclercq N, Vereecken NJ (2020). Managed honey bees as a radar for wild bee decline?. Apidologie.

[CR92] Woodcock BA, Isaac NJB, Bullock JM, Roy DB, Garthwaite DG, Crowe A, Pywell RF (2016). Impacts of neonicotinoid use on long-term population changes in wild bees in England. Nat Commun.

