# Supplementary material for: A new exposure protocol adapted for wild bees reveals species-specific impacts of the sulfoximine insecticide sulfoxaflor
Source: Ecotoxicology. 2024 Apr 22;33(6):546–59. doi: 10.1007/s10646-024-02750-2 (PMC11252182; doi:10.1007/s10646-024-02750-2)
Supplement: Supplementary file 1 — Supplementary Information [file 10646_2024_2750_MOESM1_ESM.docx]

**A new exposure protocol adapted for wild bees reveals species-specific impacts of the sulfoximine insecticide sulfoxaflor**

Justine Dewaele*^1,2^**^†^**, Alexandre Barraud^1^**^†^**, Sara Hellström^3^, Robert J. Paxton^3^, Denis Michez^1^

*^1^University of Mons (UMons), Research institute for Biosciences, Laboratory of Zoology, Place du Parc 20, 7000 Mons, Belgium.*

*^2^University of Lille, CNRS, UMR 8198 – Evo-Eco-Paleo, FR-59000 Lille, France*.

*^3^Martin Luther University Halle-Wittenberg, General Zoology, Institute for Biology, Hoher Weg 8, 06120, Halle, Germany*

^*^**Corresponding authors**: [justine.dewaele@umons.ac.be](mailto:justine.dewaele@umons.ac.be); **^†^** These authors equally contributed to this work.

**Table S1 – Adjustments to OECD guidelines for testing chemicals on bumblebee, acute oral toxicity test, n°247.** Legend: “-“ = no adjustment required, “none”= no adjustment made.

| OECD protocol paragraph | Line | Original content | Adjustments |
| --- | --- | --- | --- |
| Introduction | 1-3 | - | - |
| Initial considerations and limitations | 4 | - | - |
|  | 5 | Detailing of the aim of the test guideline that is mainly the determination of the acute oral exposure LD50 of bumblebee workers. | In this adapted protocol, our aim is to avoid the evaluation of the LD50 for each species as a limited number of individuals was available therefore limiting the number of possible replicates. Instead, the aim is to compare the sensitivity of wild bee species to *B. terrestris*, by exposing them orally to one lethal dose (>LD50). |
| Principle of the test | 6 | Adult worker bumblebees exposed to 50% aqueous sucrose solution containing the test chemical. The test duration is 48h and can be extended up to 96h if the mortality rate increases by ≥ 10% between 24h and 48h while the control mortality at ≤ 10%. The mortality has to be recorded daily and compared with control values. The results are used to calculate LD50 and NOED. | Here, wild bees were exposed and the test stopped after 48h. Results were analysed in order to compare wild bee sensitivity to one dose to the sensitivity of *B. terrestris.* |
| Validity of the test | 7 | - | - |
| Description of the method – Test organism | 8 | Adult bumblebee workers (Bombus spp.) | Adult females of wild-caught bees. |
|  | 9 | Medium sized bumblebee colonies presenting brood at all stages of development and a laying queen are used. | Females of wild bees are caught in the wild at the entrance of the nest when possible, or while foraging, with a cotton insect net. Whenever possible, we captured them at the beginning of their flying season to ensure young female testing. |
| Description of the method – Test cages | 10 | Single housing is used as it prevents competition fights potentially introducing mortality. The bumblebees are fed individually. | none |
|  | 11 | Cages are used and can be of the following material stainless steel, cardboard, wire mesh, wire mesh, plastic, wooden cages, etc. The nicot cages are the recommended format for bumblebees. | Plastic beakers are used allowing bigger space for the wild bees to move more freely. |
|  | 12 | - | - |
| Description of the method – Collection and Randomization of bumblebees | 13 | Adult bumblebees are collected from the colony either under red light or anesthetized by chilling. Only standard sized bumblebees are used. Newly emerged workers, drones and queens are not to be used. | The same procedure was used for *Bombus* species. For wild bees, they were caught directly at the entrance of the nest and while foraging on flowers. Very small and particularly very large bees were excluded from the test by visual inspection. |
|  | 14 | The individuals are individually weighed and randomly allocated to the treatment groups. | none |
| Description of the method – Handling and feeding | 15 | The individuals are manipulated under red or artificial light. The untreated feeding solution is a 50% dilution of sucrose into water and is given *ad libitum* to the workers during the whole duration of the experiment though a plastic syringe preferably with the tip removed. | During the observation periods, feeding solutions were offered to the bees using soaked cotton capillaries renewed every day. During the exposure period, the contaminated feeding solutions were offered through spectrophotometry cuvettes. Wild bees feed with difficulties from commercially available plastic syringes and they were avoided. |
| Description of the method – Preparation of the test organism | 16 | Bumblebees are acclimatised to the test conditions during at least 8h with ad libitum untreated feeding solution. Moribund bumblebees are discarded and replaced before starting the test. That is why 5% more bumblebees than the total number planned for the test are to be installed at the beginning. | Bees were acclimatised to the test conditions (including single housing) for 12h with access to an untreated 50 % (w/v) aqueous sucrose solution ad libitum. When moribund bees occurred, these were discarded. It was not always possible to replace moribund individuals by healthy ones due to the low number of individuals caught in the wild. |
|  | 17 | A starvation period of 2 to 4h was respected before the exposure period to ensure that the diet will be entirely consumed within max. 4h. | The starvation period is longer in wild bees for which four hours was the minimum required starvation period as wild bees rarely make stock of nectar in their nest contrarily to bumblebees and are therefore adapted to longer starvation periods. |
| Description of the method – Preparation of the tested doses | 18-20 | The test chemical is prepared either by first preparing a stock solution or by directly diluting the chemical into the sucrose-water feeding solution. When the chemical can be easily diluted into water, water is used as solvent. If the chemical is not easily soluble in water, other solvent can be used with the proper control group. If acetone is used, the concentration must not exceed 5%. | none |
| Description of the method – Analytical Verification | 21-22 | - | - |
| Test procedure – Test and control groups | 23 | Detailing the statistical requirements needed in terms of number of doses and replicates for the determination of LD50 with 95% confidence limits. | not applicable because no LD50 nor dose-effect curve establishments. |
|  | 24 | The minimum required replicates (cages) by treatment group must be 30. | The number of replicates (individual tested) depended on the number of individuals caught in the wild. |
|  | 25 | - | - |
| Test procedure – Treatment of controls when a solvent is used | 26 | If solvent is used, a control group treated with the solvent must be installed in addition to the water control group. | Due to the low number of individual available, we only prepared one control solution containing the solvent. |
| Test procedure – Reference substance | 27 | A dose of a reference substance leading to a mortality rate higher than 50% should be used. | Due to the low number of individual available for wild bee species, we only treated a group of 30 *Bombus terrestris* workers with dimethoate as reference substance. |
| Test procedure – Exposure (feeding) | 28 | The volume of treated or control solution given to each bumblebee was 40µL. The syringes are weighed before and after the exposure period to determine the amount consumed. The duration of the exposure period cannot exceed 4h. After the exposure period or when the worker has consumed the entirety of the solution, the untreated diet is replaced and given back *ad libitum.* | Each bee was provided with 20µL of treated or control solution instead of 40µL to ensure appropriate feeding. The consumption of the whole volume was checked visually every 30 minutes. |
|  | 29 | - | - |
|  | 30-32 | Non-feeder can occur and need either to be considered or to be replaced. In order to be replaced, more individual than the number entering the test should be installed. A non-feeder is an individual that consumes less than 80% of the solution. They will not be considered in the experiment. The non-feeders can be recorded to record repellent effect of the tested chemical. | When non-feeders occurred, we discarded them from the test. The non-feeders and the feeders occurring in the control groups were recorded and the data were used in the feeding ability test. |
| Test procedure – Test conditions | 33 | The test conditions include a constant darkness when not observed or handled, a temperature of 25±2°C and a relative humidity of 60±20%. | During the whole experiment, the wild bees were kept under natural daylight and at room temperature (around 21°C). |
| Test procedure - Duration | 34 | The observation duration is 48h and can be extended up to 96h if the mortality rate increases by ≥ 10% between 24h and 48h while the control mortality at ≤ 10%. | The observation period stopped after 48hours. |
| Test procedure – Observations and measurements | 35 | Mortality is recorded after the end of the exposure period and after 24 and 48h. If the observation session is prolonged, the mortality has to be checked after 72h and 96h. | Mortality was recorded within 16h after start of the test chemical administration as well as after 24 h and 48 h. |
|  | 36 | The recording of the sub-lethal effects should be made daily and recorded as “unaffected”, “affected” and moribund”. | none |
| Limit test | 37-38 | Explanation of the conduction of a limit test before starting the acute oral exposure experiment. | no limit test was done here because a single dose was tested. |
| Data and data reporting – Data treatment | 39 | - | - |
| Data and data reporting – Test report | 40 | - | - |

**Table S2 – Adjustments to OECD guidelines for testing chemicals on bumblebee, acute contact toxicity test, n°247.** Legend: “-“ = no adjustment possible, “same”= no adjustment made.

| OECD protocol paragraph | Line | Original content | Adjustments |
| --- | --- | --- | --- |
| Introduction | 1-3 | - | - |
| Initial considerations and limitations | 4 | - | - |
|  | 5 | Detailing of the aim of the test guideline that is mainly the determination of the acute contact exposure LD50 of bumblebee workers. | In this adapted protocol, our aim is to avoid the evaluation of the LD50 for each species as a limited number of individuals was available therefore limiting the number of possible replicates. Instead, the aim is to compare the sensitivity of wild bee species to *B. terrestris*, by exposing them topicaly to one lethal dose (>LD50). |
| Principle of the test | 6 | Adult worker bumblebees exposed to a test chemical in an appropriate carrier through a droplet deposited on the dorsal part of the thorax. The test duration is 48h and can be extended up to 96h if the mortality rate increases by ≥ 10% between 24h and 48h while the control mortality at ≤ 10%. The mortality has to be recorded daily and compared with control values. The results are used to calculate LD50 and NOED. | Here, wild bees were exposed and the test stopped after 48h. Results were analysed in order to compare wild bee sensitivity to one dose to the sensitivity of *B. terrestris.* |
| Validity of the test | 7 | - | - |
| Description of the method – Test organism | 8 | Adult bumblebee workers (Bombus spp.) | Adult females of wild-caught bees. |
|  | 9 | Medium sized bumblebee colonies presenting brood at all stages of development and a laying queen are used. | Females of wild bees are caught in the wild at the entrance of the nest when possible, or while foraging, with a cotton insect net. Whenever possible, we captured them at the beginning of their flying season to ensure young female testing. |
| Description of the method – Test cages | 10 | Single housing is used as it prevents competition fights potentially introducing mortality. The bumblebees are fed individually. | none |
|  | 11 | Cages are used and can be of the following material stainless steel, cardboard, wire mesh, wire mesh, plastic, wooden cages, etc. The nicot cages are the recommended format for bumblebees. | Plastic beakers are used allowing bigger space for the wild bees to move more freely. |
|  | 12 | - | - |
| Description of the method – Collection and randomization of bumblebees | 13 | Adult bumblebees are collected from the colony either under red light or anesthetized by chilling. Only standard sized bumblebees are used. Newly emerged workers, drones and queens are not to be used. | The same procedure was used for *Bombus* species. For wild bees, they were caught directly at the entrance of the nest and while foraging on flowers. Very small and particularly very large bees were excluded from the test by visual inspection. |
|  | 14 | The individuals are individually weighed and randomly allocated to the treatment groups. | none |
| Description of the method – Handling and feeding | 15 | The individuals are manipulated under red or artificial light. The untreated feeding solution is a 50% dilution of sucrose into water and is given *ad libitum* to the workers during the whole duration of the experiment though a plastic syringe preferably with the tip removed. | During the observation periods, feeding solutions were offered to the bees using soaked cotton capillaries renewed every day. Wild bees feed with difficulties from commercially available plastic syringes and they were avoided. |
| Description of the method – Preparation of the test organism | 16 | Bumblebees are acclimatised to the test conditions during at least 8h with ad libitum untreated feeding solution. Moribund bumblebees are discarded and replaced before starting the test. That is why 5% more bumblebees than the total number planned for the test are to be installed at the beginning. | Bees were acclimatised to the test conditions (including single housing) for 12h with access to an untreated 50 % (w/v) aqueous sucrose solution ad libitum. When moribund bees occurred, these were discarded. It was not always possible to replace moribund individuals by healthy ones due to the low number of individuals caught in the wild. |
| Description of the method – Preparation of the test doses | 17 | An organic solvent or a water solution with proper control group installation can be used as carrier to apply the test chemical. | none |
|  | 18 | Control group containing the solvent or surfactant used should be installed and treated. | Due to the low number of individual available, we only prepared one control solution containing the solvent and the surfactant. |
| Description of the method – Analytical verification | 19-20 | - | - |
| Test procedure – Test and control groups | 21 | Detailing the statistical requirements needed in terms of number of doses and replicates for the determination of LD50 with 95% confidence limits. | Not applicable because no LD50 and dose-effect curve establishments |
|  | 22 | The minimum required replicates (cages) by treatment group must be 30. | The number of replicates (individual tested) depended on the number of individuals caught in the wild. |
|  | 23 | - | - |
| Test procedure – Treatment of controls when a solvent is used | 24 | If solvent is used, a control group treated with the solvent must be installed in addition to the water control group. | Due to the low number of individual available, we only prepared one control solution containing the solvent. |
| Test procedure – Reference substance | 25 | A dose of a reference substance leading to a mortality rate higher than 50% should be used. | Due to the low number of individual available for wild bee species, we only treated a group of 30 *Bombus terrestris* workers with dimethoate as reference substance. |
| Test procedure – Exposure | 26 | The bumblebees are anesthetized either by chilling or with CO2 before being handled for topical exposure. A drop of 2µL containing either the test or control solution is deposited on the hairless part of the thorax. The workers are replaced in their cages with ad libitum untreated diet for the remaining of the experiment. | The bees were placed in a petri dish to be chilled. |
| Test procedure – Test conditions | 27 | The test conditions include a constant darkness when not observed or handled, a temperature of 25±2°C and a relative humidity of 60±20%. | During the whole experiment, the wild bees were kept under natural daylight and at room temperature (around 21°C). |
| Test procedure - Duration | 28 | The observation duration is 48h and can be extended up to 96h if the mortality rate increases by ≥ 10% between 24h and 48h while the control mortality at ≤ 10%. | The observation period stopped after 48hours |
| Test procedure – Observations and measurements | 29 | Mortality is recorded after the end of the exposure period and after 24 and 48h. If the observation session is prolonged, the mortality has to be checked after 72h and 96h. | Mortality was recorded within 16h after start of the test chemical administration as well as after 24 h and 48 h. |
|  | 30 | The recording of the sub-lethal effects should be made daily and recorded as “unaffected”, “affected” and moribund”. | none |
| Limit test | 31 | Explanation of the conduction of a limit test before starting the acute contact exposure experiment. | no limit test weas done here because a single dose was tested. |
| Data and reporting – Data treatment | 32 | - | - |
| Data and data reporting – Test report | 33 | - | - |

**Table S3 – Details about doses used for the oral exposure with information of mean weight compared to the published endpoints.**

| Species | Sulfoxaflor group average fresh weight  ± SE (g) | Specific averaged oral doses | | Published LD50 (µg/bee) | |
| --- | --- | --- | --- | --- | --- |
|  |  | as µg/ g b.w. | as µg/bee | Linguadoca et al., 2022 | EFSA, 2014 |
| *B. terrestris* | 0.261 ± 0.003 | 0.563 | 0.262 ± 0.062 | 0.126 | 0.027 |
| *B. hypnorum* | 0.148 ± 0.042 | 0.563 | 0.083 ± 0.024 |  |  |
| *B. pascuorum* | 0.172 ± 0.034 | 0.563 | 0.097 ± 0.019 |  |  |
| *A. vaga* | 0.138 ± 0.023 | 0.563 | 0.078 ± 0.013 |  |  |
| *H. scabiosae* | 0.088 ± 0.020 | 0.563 | 0.050 ± 0.011 |  |  |
| *O. cornuta* | 0.122 ± 0.002 | 0.563 | 0.068 ± 0.009 |  |  |

**Table S4 – Details about doses used for the topical exposure with information of mean weight compared to the published endpoints.**

| Species | Average fresh weight  ± SE (g) | Specific averaged topical dose | Published LD50 (µg/bee) | |
| --- | --- | --- | --- | --- |
|  |  | as µg/bee | Linguadoca et al., 2022 | EFSA, 2014 |
| *B. terrestris* | 0.259 ± 0.057 | 10.4 | 6.322 | 7.55 |
| *B. hypnorum* | 0.163 ± 0.060 | 10.4 |  |  |
| *B. pascuorum* | 0.135 ± 0.031 | 10.4 |  |  |
| *A. plumipes* | 0.127 ± 0.012 | 10.4 |  |  |
| *A. vaga* | 0.134 ± 0.023 | 10.4 |  |  |
| *H. truncorum* | 0.014 ± 0.003 | 10.4 |  |  |
| *O. leaiana* | 0.070 ± 0.024 | 10.4 |  |  |
| *O. caerulescens* | 0.043 ± 0.007 | 10.4 |  |  |

**Table S5 – Results of the positive control experiment with dimethoate topical (10µg/bee) and oral (4µg/bee) exposure of *Bombus terrestris* with the same exposure protocol as used in the wild bee design**. (GLMM with binomial family the initial colony with as random factor, for the oral exposure, as complete separation occurred, we used a Bayesian GLMM with weak prior). Significance level: ***p<0.001.

| Exposure protocol | Treatments | Probability of mortality (95% CI) | χ² | Df. | p-value |
| --- | --- | --- | --- | --- | --- |
| Oral | Control | 0.04 (0.01, 0.19) | 29.16 | 1 | 6.667e-08 *** |
|  | Dimethoate | 0.96 (0.82, 0.99) |  |  |  |
| Topical | Control | 0.03 (0.00, 0.18) | 26.91 | 1 | 2.127e-07 *** |
|  | Dimethoate | 0.95 (0.82, 0.99) |  |  |  |


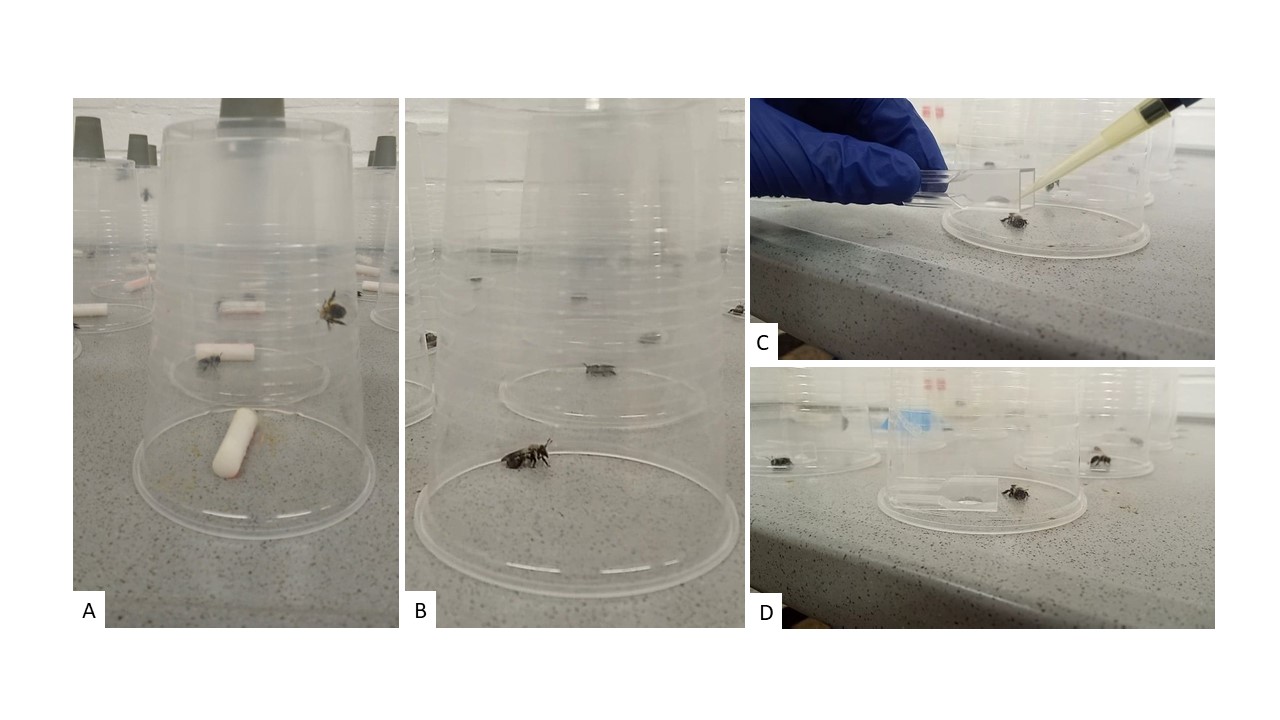


**Fig. S1 - Example of acute oral exposure to sulfoxaflor with *Andrena vaga*.** A) Acclimation period under a see-through plastic glass with a soaked capillary as food source, B) starvation period with the soak capillary removed from the glass, C) positioning of the 20μL droplet inside the spectrophotometer cuvette, and D) exposure period with the cuvettes under the glass.


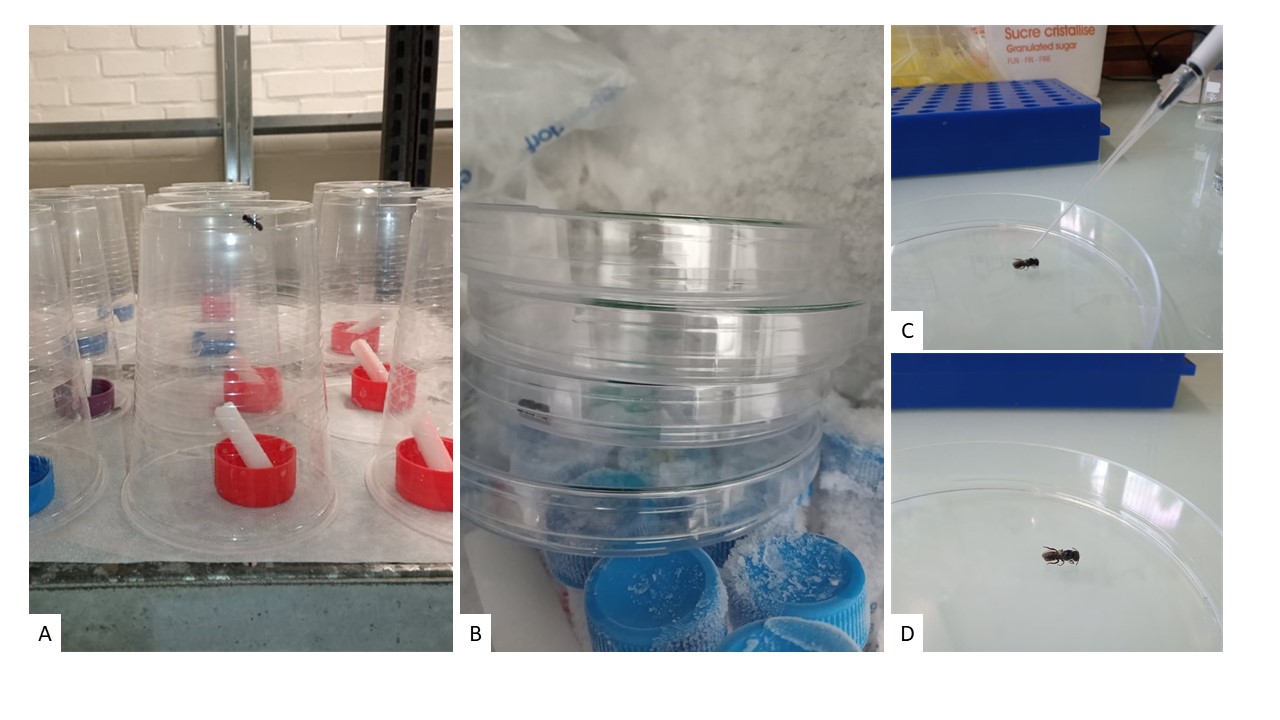


**Fig. S2 - Example of acute topical exposure to sulfoxaflor with Heriades truncorum.** A) Acclimation period under a see-through plastic glass with a soaked capillary as food source, B) chilling process into petri dishes, C) topical exposure by positioning a 2μL droplet on the bee thorax, and D) recovery period into the petri dish after chilling and before going back under the see-through plastic glass.

**Table S6 – Statistical results of the Bayesian GLMM with binomial family used to evaluate the effects of oral exposure to sulfoxaflor (0.563 μg/g body weight) and of topical exposure to sulfoxaflor (10.4µg/bee) on the mortality rate and the difference in sensitivity of the tested wild bee species with *Bombus terrestris*.** Significance level: ***p<0.001.

| Exposure protocol | Terms | χ² | Df. | p-value |
| --- | --- | --- | --- | --- |
| Oral | Treatment | 64.865 | 1 | 8.022e-16 *** |
|  | Species | 66.097 | 5 | 6.635e-13 *** |
|  | Treatment:Species | 23.741 | 5 | 0.0002434 *** |
| Topical | Treatment | 232.026 | 1 | < 2.2e-16 *** |
|  | Species | 64.978 | 7 | 1.519e-11 *** |
|  | Treatment:Species | 10.842 | 7 | 0.1457 |

**Table S7 – Detailed results of the pairwise comparison after the Bayesian GLMM with binomial family used to evaluate the effects of oral exposure to sulfoxaflor (0.563 μg/g body weight) on the mortality rate and the difference in sensitivity of the tested wild bee species with *Bombus terrestris*.** Grey cells show contrast between control and treatment of the same species. Bold text in cell show contrast between wild bee treatment group and *B. terrestris*. Significance level: *p<0.05; **p<0.01; ***p<0.001.

| contrast | estimate | SE | p.value |
| --- | --- | --- | --- |
| control A. vaga - sulfoxaflor A. vaga | -1.2491 | 0.358 | 0.0019** |
| control A. vaga - control B. hypnorum | 1.7364 | 0.696 | 0.0298* |
| control A. vaga - sulfoxaflor B. hypnorum | 2.0494 | 0.883 | 0.0433* |
| control A. vaga - control B. pascuorum | -0.1765 | 0.572 | 0.8063 |
| control A. vaga - sulfoxaflor B. pascuorum | -1.4676 | 0.540 | 0.0160* |
| control A. vaga - control B. terrestris | 3.2422 | 1.053 | 0.0062** |
| control A. vaga - sulfoxaflor B. terrestris | -1.2980 | 0.363 | 0.0015** |
| control A. vaga - control H. scabiosae | 0.9087 | 1.087 | 0.4766 |
| control A. vaga - sulfoxaflor H. scabiosae | -1.4143 | 0.835 | 0.1386 |
| control A. vaga - control O. cornuta | -0.8228 | 0.472 | 0.1272 |
| control A. vaga - sulfoxaflor O. cornuta | -2.7935 | 0.683 | 0.0003*** |
| sulfoxaflor A. vaga - control B. hypnorum | 2.9855 | 0.699 | 0.0002*** |
| sulfoxaflor A. vaga - sulfoxaflor B. hypnorum | 3.2985 | 0.848 | 0.0006*** |
| sulfoxaflor A. vaga - control B. pascuorum | 1.0726 | 0.573 | 0.1012 |
| sulfoxaflor A. vaga - sulfoxaflor B. pascuorum | -0.2185 | 0.498 | 0.7396 |
| sulfoxaflor A. vaga - control B. terrestris | 4.4913 | 1.061 | 0.0002*** |
| ***sulfoxaflor A. vaga - sulfoxaflor B. terrestris*** | ***-0.0489*** | ***0.318*** | ***0.8991*** |
| sulfoxaflor A. vaga - control H. scabiosae | 2.1578 | 1.120 | 0.0938 |
| sulfoxaflor A. vaga - sulfoxaflor H. scabiosae | -0.1652 | 0.805 | 0.8772 |
| sulfoxaflor A. vaga - control O. cornuta | 0.4263 | 0.457 | 0.4451 |
| sulfoxaflor A. vaga - sulfoxaflor O. cornuta | -1.5444 | 0.642 | 0.0367* |
| control B. hypnorum - sulfoxaflor B. hypnorum | 0.3130 | 0.997 | 0.8063 |
| control B. hypnorum - control B. pascuorum | -1.9130 | 0.832 | 0.0442* |
| control B. hypnorum - sulfoxaflor B. pascuorum | -3.2040 | 0.793 | 0.0004*** |
| control B. hypnorum - control B. terrestris | 1.5058 | 1.218 | 0.2975 |
| control B. hypnorum - sulfoxaflor B. terrestris | -3.0344 | 0.690 | 0.0002*** |
| control B. hypnorum - control H. scabiosae | -0.8277 | 1.262 | 0.5827 |
| control B. hypnorum - sulfoxaflor H. scabiosae | -3.1507 | 1.017 | 0.0061** |
| control B. hypnorum - control O. cornuta | -2.5593 | 0.760 | 0.0028** |
| control B. hypnorum - sulfoxaflor O. cornuta | -4.5300 | 0.894 | <.0001*** |
| sulfoxaflor B. hypnorum - control B. pascuorum | -2.2260 | 0.975 | 0.0449* |
| sulfoxaflor B. hypnorum - sulfoxaflor B. pascuorum | -3.5170 | 0.937 | 0.0009*** |
| sulfoxaflor B. hypnorum - control B. terrestris | 1.1928 | 1.322 | 0.4484 |
| ***sulfoxaflor B. hypnorum - sulfoxaflor B. terrestris*** | ***-3.3474*** | ***0.852*** | ***0.0005****** |
| sulfoxaflor B. hypnorum - control H. scabiosae | -1.1407 | 1.368 | 0.4766 |
| sulfoxaflor B. hypnorum - sulfoxaflor H. scabiosae | -3.4637 | 1.131 | 0.0063** |
| sulfoxaflor B. hypnorum - control O. cornuta | -2.8723 | 0.912 | 0.0054** |
| sulfoxaflor B. hypnorum - sulfoxaflor O. cornuta | -4.8430 | 1.022 | <.0001*** |
| control B. pascuorum - sulfoxaflor B. pascuorum | -1.2910 | 0.662 | 0.0915 |
| control B. pascuorum - control B. terrestris | 3.4188 | 1.151 | 0.0080** |
| control B. pascuorum - sulfoxaflor B. terrestris | -1.1215 | 0.562 | 0.0853 |
| control B. pascuorum - control H. scabiosae | 1.0853 | 1.198 | 0.4484 |
| control B. pascuorum - sulfoxaflor H. scabiosae | -1.2377 | 0.935 | 0.2607 |
| control B. pascuorum - control O. cornuta | -0.6463 | 0.646 | 0.4107 |
| control B. pascuorum - sulfoxaflor O. cornuta | -2.6170 | 0.799 | 0.0037** |
| sulfoxaflor B. pascuorum - control B. terrestris | 4.7098 | 1.125 | 0.0002*** |
| ***sulfoxaflor B. pascuorum - sulfoxaflor B. terrestris*** | ***0.1696*** | ***0.498*** | ***0.8063*** |
| sulfoxaflor B. pascuorum - control H. scabiosae | 2.3763 | 1.177 | 0.0845 |
| sulfoxaflor B. pascuorum - sulfoxaflor H. scabiosae | 0.0533 | 0.896 | 0.9526 |
| sulfoxaflor B. pascuorum - control O. cornuta | 0.6447 | 0.594 | 0.3737 |
| sulfoxaflor B. pascuorum - sulfoxaflor O. cornuta | -1.3260 | 0.753 | 0.1258 |
| control B. terrestris - sulfoxaflor B. terrestris | -4.5402 | 1.049 | 0.0002*** |
| control B. terrestris - control H. scabiosae | -2.3335 | 1.491 | 0.1762 |
| control B. terrestris - sulfoxaflor H. scabiosae | -4.6565 | 1.292 | 0.0014** |
| control B. terrestris - control O. cornuta | -4.0650 | 1.100 | 0.0010** |
| control B. terrestris - sulfoxaflor O. cornuta | -6.0357 | 1.198 | <.0001*** |
| sulfoxaflor B. terrestris - control H. scabiosae | 2.2067 | 1.109 | 0.0853 |
| ***sulfoxaflor B. terrestris - sulfoxaflor H. scabiosae*** | ***-0.1163*** | ***0.807*** | ***0.8991*** |
| sulfoxaflor B. terrestris - control O. cornuta | 0.4752 | 0.447 | 0.3793 |
| ***sulfoxaflor B. terrestris - sulfoxaflor O. cornuta*** | ***-1.4955*** | ***0.645*** | ***0.0433**** |
| control H. scabiosae - sulfoxaflor H. scabiosae | -2.3230 | 1.220 | 0.0963 |
| control H. scabiosae - control O. cornuta | -1.7316 | 1.151 | 0.1943 |
| control H. scabiosae - sulfoxaflor O. cornuta | -3.7023 | 1.248 | 0.0080* |
| sulfoxaflor H. scabiosae - control O. cornuta | 0.5914 | 0.870 | 0.5749 |
| sulfoxaflor H. scabiosae - sulfoxaflor O. cornuta | -1.3793 | 0.985 | 0.2315 |
| control O. cornuta - sulfoxaflor O. cornuta | -1.9707 | 0.701 | 0.0125* |

**Table S8 – Detailed results of the pairwise comparison after the Bayesian GLMM with binomial family used to evaluate the effects of topical exposure to sulfoxaflor (10.4µg/bee) on the mortality rate and the difference in sensitivity of the tested wild bee species with *Bombus terrestris*.** Grey cells show contrast between control and treatment of the same species. Bold text in cell show contrast between wild bee treatment group and *B. terrestris*. Significance level: *p<0.05; **p<0.01; ***p<0.001.

| contrast | estimate | SE | p.value |
| --- | --- | --- | --- |
| control A. plumipes - sulfoxaflor A. plumipes | -3.1505 | 0.564 | <.0001*** |
| control A. plumipes - control A. vaga | 2.2726 | 0.646 | 0.0015** |
| control A. plumipes - sulfoxaflor A. vaga | -1.8735 | 0.483 | 0.0005*** |
| control A. plumipes - control B. hypnorum | 0.9975 | 0.733 | 0.2369 |
| control A. plumipes - sulfoxaflor B. hypnorum | -1.1648 | 0.672 | 0.1264 |
| control A. plumipes - control B. pascuorum | 0.0212 | 0.395 | 0.9729 |
| control A. plumipes - sulfoxaflor B. pascuorum | -4.1748 | 0.892 | <.0001*** |
| control A. plumipes - control B. terrestris | 1.7421 | 0.469 | 0.0009*** |
| control A. plumipes - sulfoxaflor B. terrestris | -1.1014 | 0.393 | 0.0119* |
| control A. plumipes - control H. truncorum | 1.0722 | 0.612 | 0.1247 |
| control A. plumipes - sulfoxaflor H. truncorum | -4.3628 | 1.521 | 0.0102* |
| control A. plumipes - control O. caerulescens | -0.0565 | 0.646 | 0.965 |
| control A. plumipes - sulfoxaflor O. caerulescens | -4.2892 | 1.646 | 0.0186* |
| control A. plumipes - control O. leaiana | 1.4539 | 1.147 | 0.2704 |
| control A. plumipes - sulfoxaflor O. leaiana | -3.0835 | 1.672 | 0.1056 |
| sulfoxaflor A. plumipes - control A. vaga | 5.4231 | 0.825 | <.0001*** |
| sulfoxaflor A. plumipes - sulfoxaflor A. vaga | 1.277 | 0.645 | 0.0806 |
| sulfoxaflor A. plumipes - control B. hypnorum | 4.148 | 0.907 | <.0001*** |
| sulfoxaflor A. plumipes - sulfoxaflor B. hypnorum | 1.9857 | 0.78 | 0.021* |
| sulfoxaflor A. plumipes - control B. pascuorum | 3.1717 | 0.61 | <.0001*** |
| sulfoxaflor A. plumipes - sulfoxaflor B. pascuorum | -1.0243 | 0.947 | 0.3457 |
| sulfoxaflor A. plumipes - control B. terrestris | 4.8926 | 0.673 | <.0001*** |
| **sulfoxaflor A. plumipes - sulfoxaflor B. terrestris** | **2.0491** | **0.588** | **0.0016**** |
| sulfoxaflor A. plumipes - control H. truncorum | 4.2227 | 0.779 | <.0001*** |
| sulfoxaflor A. plumipes - sulfoxaflor H. truncorum | -1.2123 | 1.526 | 0.5074 |
| sulfoxaflor A. plumipes - control O. caerulescens | 3.094 | 0.81 | 0.0006*** |
| sulfoxaflor A. plumipes - sulfoxaflor O. caerulescens | -1.1387 | 1.619 | 0.5561 |
| sulfoxaflor A. plumipes - control O. leaiana | 4.6044 | 1.278 | 0.0012** |
| sulfoxaflor A. plumipes - sulfoxaflor O. leaiana | 0.067 | 1.665 | 0.9729 |
| control A. vaga - sulfoxaflor A. vaga | -4.1461 | 0.687 | <.0001*** |
| control A. vaga - control B. hypnorum | -1.2751 | 0.91 | 0.2247 |
| control A. vaga - sulfoxaflor B. hypnorum | -3.4374 | 0.846 | 0.0003*** |
| control A. vaga - control B. pascuorum | -2.2514 | 0.652 | 0.0018** |
| control A. vaga - sulfoxaflor B. pascuorum | -6.4474 | 1.026 | <.0001*** |
| control A. vaga - control B. terrestris | -0.5305 | 0.701 | 0.5288 |
| control A. vaga - sulfoxaflor B. terrestris | -3.374 | 0.647 | <.0001*** |
| control A. vaga - control H. truncorum | -1.2004 | 0.809 | 0.1968 |
| control A. vaga - sulfoxaflor H. truncorum | -6.6354 | 1.602 | 0.0002*** |
| control A. vaga - control O. caerulescens | -2.3291 | 0.837 | 0.0125* |
| control A. vaga - sulfoxaflor O. caerulescens | -6.5618 | 1.72 | 0.0006*** |
| control A. vaga - control O. leaiana | -0.8187 | 1.276 | 0.5956 |
| control A. vaga - sulfoxaflor O. leaiana | -5.3561 | 1.748 | 0.0063** |
| sulfoxaflor A. vaga - control B. hypnorum | 2.8709 | 0.795 | 0.0012** |
| sulfoxaflor A. vaga - sulfoxaflor B. hypnorum | 0.7086 | 0.708 | 0.3843 |
| sulfoxaflor A. vaga - control B. pascuorum | 1.8946 | 0.469 | 0.0003*** |
| sulfoxaflor A. vaga - sulfoxaflor B. pascuorum | -2.3013 | 0.912 | 0.0222* |
| sulfoxaflor A. vaga - control B. terrestris | 3.6156 | 0.537 | <.0001*** |
| **sulfoxaflor A. vaga - sulfoxaflor B. terrestris** | **0.7721** | **0.458** | **0.1365** |
| sulfoxaflor A. vaga - control H. truncorum | 2.9457 | 0.672 | 0.0001*** |
| sulfoxaflor A. vaga - sulfoxaflor H. truncorum | -2.4893 | 1.528 | 0.1512 |
| sulfoxaflor A. vaga - control O. caerulescens | 1.817 | 0.706 | 0.0202* |
| sulfoxaflor A. vaga - sulfoxaflor O. caerulescens | -2.4157 | 1.647 | 0.2012 |
| sulfoxaflor A. vaga - control O. leaiana | 3.3273 | 1.199 | 0.0125* |
| sulfoxaflor A. vaga - sulfoxaflor O. leaiana | -1.21 | 1.68 | 0.5491 |
| control B. hypnorum - sulfoxaflor B. hypnorum | -2.1623 | 0.876 | 0.0255* |
| control B. hypnorum - control B. pascuorum | -0.9763 | 0.747 | 0.2554 |
| control B. hypnorum - sulfoxaflor B. pascuorum | -5.1722 | 1.091 | <.0001*** |
| control B. hypnorum - control B. terrestris | 0.7447 | 0.79 | 0.4152 |
| control B. hypnorum - sulfoxaflor B. terrestris | -2.0988 | 0.744 | 0.0115* |
| control B. hypnorum - control H. truncorum | 0.0748 | 0.887 | 0.965 |
| control B. hypnorum - sulfoxaflor H. truncorum | -5.3603 | 1.645 | 0.0034** |
| control B. hypnorum - control O. caerulescens | -1.054 | 0.913 | 0.3134 |
| control B. hypnorum - sulfoxaflor O. caerulescens | -5.2867 | 1.761 | 0.0075** |
| control B. hypnorum - control O. leaiana | 0.4564 | 1.325 | 0.8043 |
| control B. hypnorum - sulfoxaflor O. leaiana | -4.081 | 1.788 | 0.0409* |
| sulfoxaflor B. hypnorum - control B. pascuorum | 1.186 | 0.66 | 0.1143 |
| sulfoxaflor B. hypnorum - sulfoxaflor B. pascuorum | -3.0099 | 1.021 | 0.0086** |
| sulfoxaflor B. hypnorum - control B. terrestris | 2.907 | 0.711 | 0.0003*** |
| **sulfoxaflor B. hypnorum - sulfoxaflor B. terrestris** | **0.0635** | **0.652** | **0.965** |
| sulfoxaflor B. hypnorum - control H. truncorum | 2.2371 | 0.817 | 0.0137* |
| sulfoxaflor B. hypnorum - sulfoxaflor H. truncorum | -3.198 | 1.595 | 0.077 |
| sulfoxaflor B. hypnorum - control O. caerulescens | 1.1083 | 0.846 | 0.2554 |
| sulfoxaflor B. hypnorum - sulfoxaflor O. caerulescens | -3.1244 | 1.708 | 0.1077 |
| sulfoxaflor B. hypnorum - control O. leaiana | 2.6187 | 1.288 | 0.073 |
| sulfoxaflor B. hypnorum - sulfoxaflor O. leaiana | -1.9187 | 1.74 | 0.3377 |
| control B. pascuorum - sulfoxaflor B. pascuorum | -4.196 | 0.863 | <.0001*** |
| control B. pascuorum - control B. terrestris | 1.7209 | 0.468 | 0.001** |
| control B. pascuorum - sulfoxaflor B. terrestris | -1.1225 | 0.378 | 0.008** |
| control B. pascuorum - control H. truncorum | 1.051 | 0.617 | 0.1329 |
| control B. pascuorum - sulfoxaflor H. truncorum | -4.384 | 1.509 | 0.0095** |
| control B. pascuorum - control O. caerulescens | -0.0777 | 0.655 | 0.965 |
| control B. pascuorum - sulfoxaflor O. caerulescens | -4.3104 | 1.631 | 0.0173* |
| control B. pascuorum - control O. leaiana | 1.4327 | 1.168 | 0.2867 |
| control B. pascuorum - sulfoxaflor O. leaiana | -3.1047 | 1.663 | 0.1017 |
| sulfoxaflor B. pascuorum - control B. terrestris | 5.9169 | 0.917 | <.0001*** |
| **sulfoxaflor B. pascuorum - sulfoxaflor B. terrestris** | **3.0734** | **0.87** | **0.0014**** |
| sulfoxaflor B. pascuorum - control H. truncorum | 5.247 | 1.001 | <.0001*** |
| sulfoxaflor B. pascuorum - sulfoxaflor H. truncorum | -0.188 | 1.691 | 0.965 |
| sulfoxaflor B. pascuorum - control O. caerulescens | 4.1183 | 1.025 | 0.0003*** |
| sulfoxaflor B. pascuorum - sulfoxaflor O. caerulescens | -0.1144 | 1.796 | 0.9729 |
| sulfoxaflor B. pascuorum - control O. leaiana | 5.6286 | 1.414 | 0.0003*** |
| sulfoxaflor B. pascuorum - sulfoxaflor O. leaiana | 1.0913 | 1.828 | 0.6232 |
| control B. terrestris - sulfoxaflor B. terrestris | -2.8435 | 0.453 | <.0001*** |
| control B. terrestris - control H. truncorum | -0.6699 | 0.67 | 0.3843 |
| control B. terrestris - sulfoxaflor H. truncorum | -6.1049 | 1.533 | 0.0003*** |
| control B. terrestris - control O. caerulescens | -1.7986 | 0.704 | 0.0209* |
| control B. terrestris - sulfoxaflor O. caerulescens | -6.0313 | 1.654 | 0.0011** |
| control B. terrestris - control O. leaiana | -0.2883 | 1.195 | 0.875 |
| control B. terrestris - sulfoxaflor O. leaiana | -4.8256 | 1.685 | 0.0102* |
| sulfoxaflor B. terrestris - control H. truncorum | 2.1736 | 0.611 | 0.0013** |
| **sulfoxaflor B. terrestris - sulfoxaflor H. truncorum** | **-3.2614** | **1.503** | **0.0531** |
| sulfoxaflor B. terrestris - control O. caerulescens | 1.0449 | 0.649 | 0.1554 |
| **sulfoxaflor B. terrestris - sulfoxaflor O. caerulescens** | **-3.1878** | **1.625** | **0.083** |
| sulfoxaflor B. terrestris - control O. leaiana | 2.5552 | 1.166 | 0.0509 |
| **sulfoxaflor B. terrestris - sulfoxaflor O. leaiana** | **-1.9821** | **1.658** | **0.2991** |
| control H. truncorum - sulfoxaflor H. truncorum | -5.435 | 1.524 | 0.0013** |
| control H. truncorum - control O. caerulescens | -1.1287 | 0.811 | 0.2261 |
| control H. truncorum - sulfoxaflor O. caerulescens | -5.3614 | 1.702 | 0.0049** |
| control H. truncorum - control O. leaiana | 0.3816 | 1.26 | 0.8313 |
| control H. truncorum - sulfoxaflor O. leaiana | -4.1557 | 1.732 | 0.0303* |
| sulfoxaflor H. truncorum - control O. caerulescens | 4.3063 | 1.6 | 0.0156* |
| sulfoxaflor H. truncorum - sulfoxaflor O. caerulescens | 0.0736 | 2.166 | 0.9729 |
| sulfoxaflor H. truncorum - control O. leaiana | 5.8167 | 1.876 | 0.0057** |
| sulfoxaflor H. truncorum - sulfoxaflor O. leaiana | 1.2793 | 2.194 | 0.6279 |
| control O. caerulescens - sulfoxaflor O. caerulescens | -4.2327 | 1.615 | 0.0181* |
| control O. caerulescens - control O. leaiana | 1.5104 | 1.278 | 0.3031 |
| control O. caerulescens - sulfoxaflor O. leaiana | -3.027 | 1.746 | 0.1264 |
| sulfoxaflor O. caerulescens - control O. leaiana | 5.7431 | 1.98 | 0.0095** |
| sulfoxaflor O. caerulescens - sulfoxaflor O. leaiana | 1.2057 | 2.272 | 0.6617 |
| control O. leaiana - sulfoxaflor O. leaiana | -4.5374 | 1.704 | 0.0166* |

**Table S9 – Mortality percentages and number of individuals comprised in each treated group of the different tested species in both oral and topical exposure experiments.**

| Experiment | Species (N total) | Treatment group | N alive | N dead | Mortality (%) |
| --- | --- | --- | --- | --- | --- |
| Oral | *B. terrestris* | Control | 91 | 0 | 0.0 |
|  |  | Sulfoxaflor | 48 | 37 | 43.5 |
|  | *B. hypnorum* | Control | 56 | 2 | 3.4 |
|  |  | Sulfoxaflor | 50 | 1 | 2.0 |
|  | *B. pascuorum* | Control | 16 | 4 | 20.0 |
|  |  | Sulfoxaflor | 10 | 9 | 47.4 |
|  | *A. vaga* | Control | 58 | 13 | 18.3 |
|  |  | Sulfoxaflor | 42 | 30 | 41.7 |
|  | *H. scabiosae* | Control | 6 | 0 | 0.0 |
|  |  | Sulfoxaflor | 3 | 3 | 50.0 |
|  | *O. cornuta* | Control | 19 | 9 | 32.1 |
|  |  | Sulfoxaflor | 3 | 11 | 78.6 |
| Topical | *B. terrestris* | Control | 61 | 7 | 10.3 |
|  |  | Sulfoxaflor | 22 | 45 | 67.2 |
|  | *B. hypnorum* | Control | 8 | 2 | 20.0 |
|  |  | Sulfoxaflor | 4 | 8 | 66.7 |
|  | *B. pascuorum* | Control | 32 | 21 | 39.6 |
|  |  | Sulfoxaflor | 1 | 56 | 98.2 |
|  | *A. plumipes* | Control | 21 | 18 | 46.2 |
|  |  | Sulfoxaflor | 3 | 38 | 92.7 |
|  | *A. vaga* | Control | 38 | 2 | 0.5 |
|  |  | Sulfoxaflor | 8 | 37 | 82.2 |
|  | *H. truncorum* | Control | 16 | 3 | 15.8 |
|  |  | Sulfoxaflor | 0 | 18 | 100.0 |
|  | *O. caerulescens* | Control | 6 | 4 | 40.0 |
|  |  | Sulfoxaflor | 0 | 11 | 100.0 |
|  | *O. leaiana* | Control | 4 | 0 | 0.0 |
|  |  | Sulfoxaflor | 0 | 4 | 100.0 |
